# SiteMotif: A graph-based algorithm for deriving structural motifs in Protein Ligand binding sites

**DOI:** 10.1371/journal.pcbi.1009901

**Published:** 2022-02-24

**Authors:** Santhosh Sankar, Nagasuma Chandra

**Affiliations:** 1 Department of Biochemistry, Indian Institute of Science, Bangalore, Karnataka, India; 2 BioSystems Science and Engineering, Indian Institute of Science, Bangalore, Karnataka, India; Max Planck Institute for Biophysical Chemistry, GERMANY

## Abstract

Studying similarities in protein molecules has become a fundamental activity in much of biology and biomedical research, for which methods such as multiple sequence alignments are widely used. Most methods available for such comparisons cater to studying proteins which have clearly recognizable evolutionary relationships but not to proteins that recognize the same or similar ligands but do not share similarities in their sequence or structural folds. In many cases, proteins in the latter class share structural similarities only in their binding sites. While several algorithms are available for comparing binding sites, there are none for deriving structural motifs of the binding sites, independent of the whole proteins. We report the development of SiteMotif, a new algorithm that compares binding sites from multiple proteins and derives sequence-order independent structural site motifs. We have tested the algorithm at multiple levels of complexity and demonstrate its performance in different scenarios. We have benchmarked against 3 current methods available for binding site comparison and demonstrate superior performance of our algorithm. We show that SiteMotif identifies new structural motifs of spatially conserved residues in proteins, even when there is no sequence or fold-level similarity. We expect SiteMotif to be useful for deriving key mechanistic insights into the mode of ligand interaction, predict the ligand type that a protein can bind and improve the sensitivity of functional annotation.

## Author summary

A large number of biological functions are orchestrated by proteins. The function of proteins is governed by its structure and its interacting ligand. However, it is known that not all residues are involved in ligand recognition. More specifically, residues that are located within 4.5 Å of ligand atoms are considered to be ’binding sites’. Here, we have developed an algorithm called SiteMotif that efficiently aligns multiple binding sites into a common frame. This process enables us to derive conservation among the binding site residues in a sequence order independent manner. The algorithm was validated extensively across five different levels and measured binding site similarities in each of them. Previous research has found multiple instances where different proteins have comparable binding sites and hence perform the same function. We present the ability of our method to detect such scenarios. Finally, As a use case, we applied SiteMotif to a set of glutathione binding proteins and derived a site based sequence motif characteristic of all glutathione binding proteins.

## Introduction

Recognising similarities and deriving relationships among protein molecules is an important task in present-day biology, often forming the basis for protein function annotation. Functional annotations are abstracted at different levels of hierarchy, such as the sequence, structure, and the binding site [1–3]. Typically a sequence based functional inference is based on an alignment of protein sequences, which works well for cases where proteins share significant sequence similarities [4]. However, there are several proteins that have no detectable sequence level similarities but show similarities in structure in the whole protein or just in the binding site and have the same function [5]. Comparison in such cases is more easily addressed by studying the three-dimensional structures of the ligand binding sites. Currently there are no established methods for automatically deriving structural motifs from protein binding sites. In this work, we seek to address this gap. Knowledge of the site motifs will (a) be useful in understanding the basis of ligand recognition, (b) enable comparison of proteins that share similarities only in their binding sites irrespective of sequence or fold level similarities and (c) facilitate prioritization of residues at the site in drug discovery applications.

Binding sites are discrete sets of atoms involving only a small number of residues which are discontinuous in sequence space, making them more challenging to compare than whole protein structures. A first step for motif identification is the structure-based superposition of the binding sites. Several site comparison methods have been developed in the last decade or so, based on matching the substructures at the binding sites, without considering sequences or folds. Some examples are ProBis, ApoC, G-LoSA and PocketAlign [6–9]. The first two require information from the whole folds for producing site alignments whereas the latter two produce site superpositions without using fold-level information. Given that the binding site of a protein is non-contiguous in sequence space, the computational complexity and time required to generate an optimal alignment is very high.

Currently, there are no established methods that derive structural motifs from a set of binding pockets using only the structural information of the pocket residues. Some of the key challenges in developing such tools are that the alignment search space is very large and requires evaluation of a large number of three-dimensional mappings. Efficient algorithms that perform well are therefore necessary. In this work, we address this need and develop a new algorithm that efficiently places multiple sites onto a common comparable framework and derives 3D motifs characteristic of that site. We rigorously validate the algorithm and demonstrate its performance in several case scenarios and derive a new site motif in glutathione binding proteins.

## Methods

### Design and implementation

#### Binding pocket definition

A predicted putative small molecular ligand binding site is hereafter referred to as ‘pocket’, while a known site from a protein-ligand complex from PDB is referred to as a ‘binding site’. In cases when the structure of the protein-ligand complex is available, the binding site is taken as a set of all residues whose atom(s) are located within 4.5Å of the ligand atom. Besides the study of known protein-ligand complexes, the usage of SiteMotif is envisaged in situations where the ligand site is unknown or the ligand itself is unknown, although the protein structure is known. In such cases, predicted binding sites, typically as consensus prediction using two or more well established pocket detection algorithms (eg., SiteHound, PocketDepth and FPocket) [10–12]. Site selection and ligand assignment can be made with the help of additional information such as known sites in evolutionarily related proteins or through a comparison to known sites that identifies geometrically and chemically similar site residues or chemically similar ligands as described before [13].

#### The Algorithmic perspective of SiteMotif

The algorithm comprises broadly of the following: (a) Distance matrix generation for each pocket and exhaustive comparison of all distance elements in each pair of pockets in the query set, (b) initial alignment seed generation, progressive seed expansion and selection of optimal seed sets, least-squares superposition guided by the seed sets to obtain the final alignment and (c) a multiple structural alignment for all pockets followed by derivation of a consensus pocket profile for each site-type.

The first module processes the pocket structure to represent each pocket residue as a set of three points (C⍺, Cβ and side chain centroid (CN) (Box 1).

**Box 1. The mathematical formulation of step 1 and step 2 of SiteMotif**

Three points representing each residue of a pocket <- {c⍺, cβ, cn} denoting c⍺, cβ and cn centroid of the side chain*

      Let S1 and S2 be the sets of points representing pockets P1 and P2 containing N1 and N2 points (residues) respectively.

Then

      For (i = 0; i< N1, i++) do

      For (j = 0; j< N1, j++) then

      S1-Total = S1[i]*S1[j] # constructing N1xN1 matrix of distances

S1-Total = (∀S1, ∀S1)*3 # Dimension of Site1

Repeat the above nested for loop for S2 and store the distances in S2-Total.

The dimension of S1-Total is N1xN1 and for S2-Total, it is N2xN2.

* For glycine, only C⍺ is considered. For alanine, Cβ is also taken as the centroid

**Fig 1:**
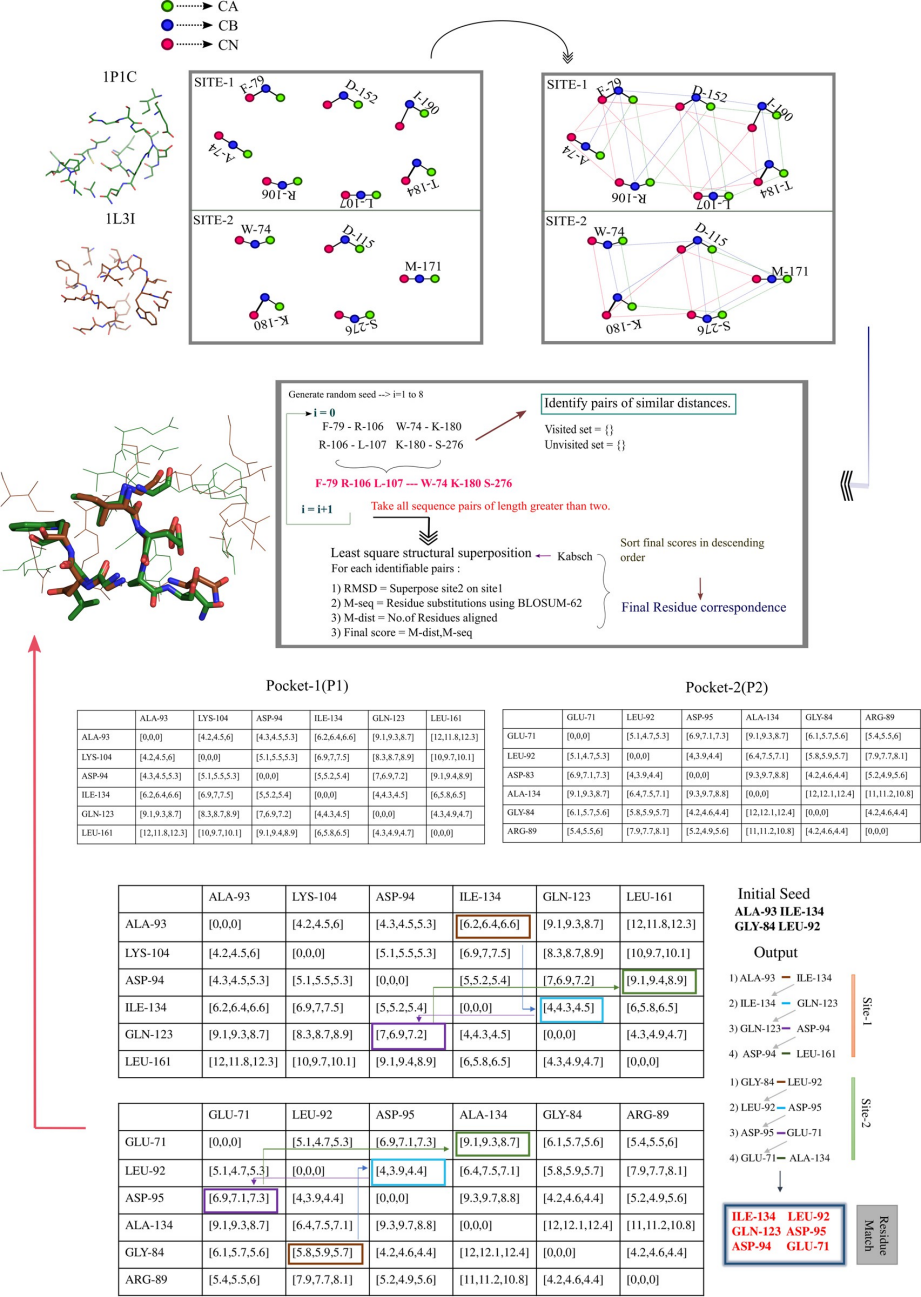
A depiction of the workflow of SiteMotif for an example pair of pockets P1 and P2. For each pocket, a distance matrix containing distances (cα-cα, cβ-cβ and cn-cn distances) for all residue pairs is constructed. For each element of P1, a scan is established against all elements of P2 and only those elements that are within 1Å to the P1 seed are selected. All matched residue pairs are then sorted in descending order of their distance difference, which will serve as the input for the next iteration. The module is run in a recursive sequence order independent manner for each element of P1 against all elements in P2. Time-Complexity at every step is reduced by creating a hash table of visited and unvisited sets which will hold information on paths that are travelled before and those yet to be traversed.

The second module generates initial seed alignments as follows. For each element in a pocket P1, SiteMotif scans the distance matrix of the second pocket P2 and chooses the closest match (within 1.0Å of the query distance element). In brief, starting from the first residue of P1, a pairwise distance R1_ij_^k^ was matched with all possible distances of R2_ij_^k^, where k represents the current distance point in the pool of distances and the subscript ij represents the matrix rows and columns of the corresponding residue list. R1 corresponds to the residue list in the first pocket (P1) and R2 represents the residue list of the second pocket (P2). If the R2_ij_^k^ distance was within the range of R1_ij_^k^ i.e their RMSD is less than 1.0Å, then take its adjacent pairs (R1_j_^k+1^) that have at least one residue match with any of the elements in j^th^ row of R2_j_. Basically a sequence order independent tree traversal is established. Subsequently, for each such matched item, it scans the distance matrix to progressively grow the seed by adding other distance elements that were equidistant to the query element in both the pockets, which results in getting a ‘seed alignment’. Although only dyad distances were considered here, in principle, it is possible to take triads, tetrads, and higher order motifs. The larger the motif, the higher the accuracy but also the higher the number of false negatives. Furthermore, larger the motif, higher the computational cost. For example, a binding site composed of 20 residues will have 380(20*19) permutations for dyads, 6840(20*19*18) permutations for triads and 11,6280(20*19*18*17) alterations for tetrads respectively, making computation difficult for triads and higher motifs. The output residue pairs from each such scan were then sorted in descending order of their distance difference. The loop was iterated for all residues in P1 against every residue in P2. The algorithm proceeds in a greedy fashion generating sequences of increasing length i.e finding common connected subgraphs in a graph (Fig 1).

It should be noted that while comparing distance elements, we may get more than one matching distance for the same seed. To overcome this, the visited matched elements were flagged as ‘selected’ and ‘not selected’ and only the latter was traversed for further matching by considering each such match as a potential seed at this stage. The seed alignments were ranked based on their lengths and the top-ranked seeds are selected for obtaining final alignments. For each shortlisted seed, SiteMotif obtains an optimal superposition between sites by performing a least squares superposition using the Kabsch algorithm [14]. Further, it queries the sequence similarity at structurally matched positions by consulting the residue substitution scores taken from the BLOSUM-62 matrix [15]. The highest scoring alignment was considered as the optimal alignment for that pair of pockets.

The faster computation time is made possible by the use of a persistent data structure (ArrayList) and hash key (visited set) which will check if a newly added candidate vertex forms a clique. If a clique is seen, then the algorithm proceeds in the same fashion adding each residue that is not present in the visited set to the persistent list until either it reaches the end of the list or when no more cliques are obtained. As a backtracking step, a newly added vertex that disturbs the clique gets removed from the ArrayList and is added to the visited set, leaving out a new list of size N-1, where N is the size of ArrayList before extraction. A three point residue representation also places additional constraints on the search space leading to exploration of fewer alignment frames and thus reducing run time.

The third module repeats the exercise for all pairs of pockets, which will result in all-pair pocket distances. SiteMotif reports three different distance-scores. M-dist_min_, M-dist_max_ corresponding to local and global structural similarities in a pair of pockets and M-seq, corresponding to the extent of similarity in the amino acids lining the pockets. M-dist_max_ is taken as the ratio of the number of matches to the total number of residues in the larger binding pocket, whereas M-dist_min_ is captured as the number of matches to the total number of residues in the smaller binding pocket. M-dist scores report similarities by counting residue matches and do not account for residue types among the aligned pairs. To address this, another scoring function M-seq score was used, which uses a scaled BLOSUM-62 matrix, where substitution values for different residue-pairs are scaled between 0 and 1. M-dist and M-seq scores range from 0 to 1, where a value of 1 indicates that the pocket pairs are identical.

To determine at what value, does the M-dist score capture pocket level similarities even at low sequence similarities, we analysed several superfamilies where there were many members belonging to the same fold but had different levels of sequence similarities. M-dist_max_ ≥ 0.4 was found to capture the known similarities correctly (S1 Fig and S1 Table). When multiple binding pockets are given as the input, the scores were calculated for each pocket-pair and a representative for the set was chosen as follows: Using all-to-all M-dist_max_ distances, the pockets were projected as a network where pockets form the nodes and the similarity between them form the edges. Clusters were identified from the network, where pocket-pairs were given membership in a given cluster of M-dist_max_ ≥ 0.4. Clustering was performed using MCODE using M-dist_max_ as the input. The pocket in each cluster that had the highest degree was chosen as the cluster representative. The cluster members were then aligned onto the representative from which a multiple site alignment was obtained. Consensus pocket residues were computed from the multiple alignments which was followed by an identification of structural motif, if any. The alignment was visualized as a sequence logo that contains information about the frequency of occurrence of each residue.

The run time performance and scalability of SiteMotif was calculated using a random set of 10,000 binding site pairs taken from the PDB. The comparison was set up on a distributed Cray system specifying the following number of processors (N: 1, 2, 4, 8, 16, 48). SiteMotif was seen to roughly perform 200 comparisons per minute on a single processor machine (S2 Fig). The execution time decreases as the number of cores increases. At N = 48, it operates at a rate of comparing 6500 pockets per minute. There are no upper limits for the CPU number as SiteMotif could run seamlessly even when specified with 2000 cores such as MPI enabled distributed clusters. All analyses were tested on a cray supercomputer using crays ‘aprun’ utility.

#### Datasets

To evaluate the performance of SiteMotif, a five-level validation scheme was designed that uses different datasets. Level-1: protein pairs that share high similarity at all levels i.e., similar sequence, similar structure, similar binding sites, and binding to the same ligand (pdb_95 dataset downloaded from RCSB-PDB, where proteins are clustered at 95% sequence identity).

Level-2: a set of protein pairs belonging to the same superfamily from the SCOP database [16] but with no significant sequence similarity (pairwise similarity using Blosum 62 < 30%) with any other protein in the set. Level-3: a set of proteins obtained from the literature based on specific reports of proteins such that they share similarity only at the binding site, i.e., no similarity is detectable at sequence and structure, Level-4: protein sets previously reported in literature of entirely different sites binding to the same ligand. Level-5: random site pairs that are totally unrelated. Levels-4 and 5 were used as negative controls.

#### Scoring scheme and clustering of sites

SiteMotif employs three scoring schemes, M-dist_min_, M-dist_max_ and M-seq to best capture local and global similarities in the binding site. M-dist_max_ was calculated by dividing the number of matches by the total number of residues in the larger binding site. Whereas, M-dist_min_ was obtained by dividing the number of matches by the total number of residues in the smaller binding site $\frac{Match(S1-Total,S2-Total)}{min(S1,S2)}$. Both M-dist scores treat residues as a set of point vectors and don’t necessarily incorporate their physicochemical properties while comparing. To account for residue substitution, M-seq was developed which estimates similarity at the residue level using BLOSUM62 matrix [17]. When multiple sites are supplied as an input, SiteMotif performs an all-vs-all comparison for all combinations of site pairs using which a projection network was constructed with Cytoscape [18]. Sites in a network are represented as nodes and edges are constructed based on SiteMotif scores. From the projection, clustering was performed using MCODE to identify representatives which serve as a template for building multiple site alignment [19].

#### Programming architecture

The Algorithm was written in python2.7 and executed on a Linux X86-64 machine. The parallel version suitable for large scale comparison was also written and implemented using mpi wrapper mpi4py [20]. The performance was tested on supercomputing clusters.

## Results

We present SiteMotif, an algorithm that compares structures of small molecule ligand binding sites from multiple proteins and derives site motifs. We describe rigorous validation of SiteMotif at different levels of complexity and also benchmark its performance against 3 current methods available for binding site comparison. We describe the use of SiteMotif for identifying new structural motifs in glutathione binding proteins.

### Sensitivity analysis

We tested the sensitivity of SiteMotif using two independent analyses, a) random perturbation of residue positions and b) random perturbation of residue types. Both the analyses were carried out based on a systematic exercise of projecting all PDB ligands into a 2D matrix representing molecular weight and the partition coefficient (LogP) of ligand, and sampled 24 distinct points from that (S6 Fig). The results of sensitivity analysis of four ligands (ATP, SAM, HEM and MTX) are briefly described below.

### a) Sensitivity with respect to random perturbation of position of residues

**Fig 2:**
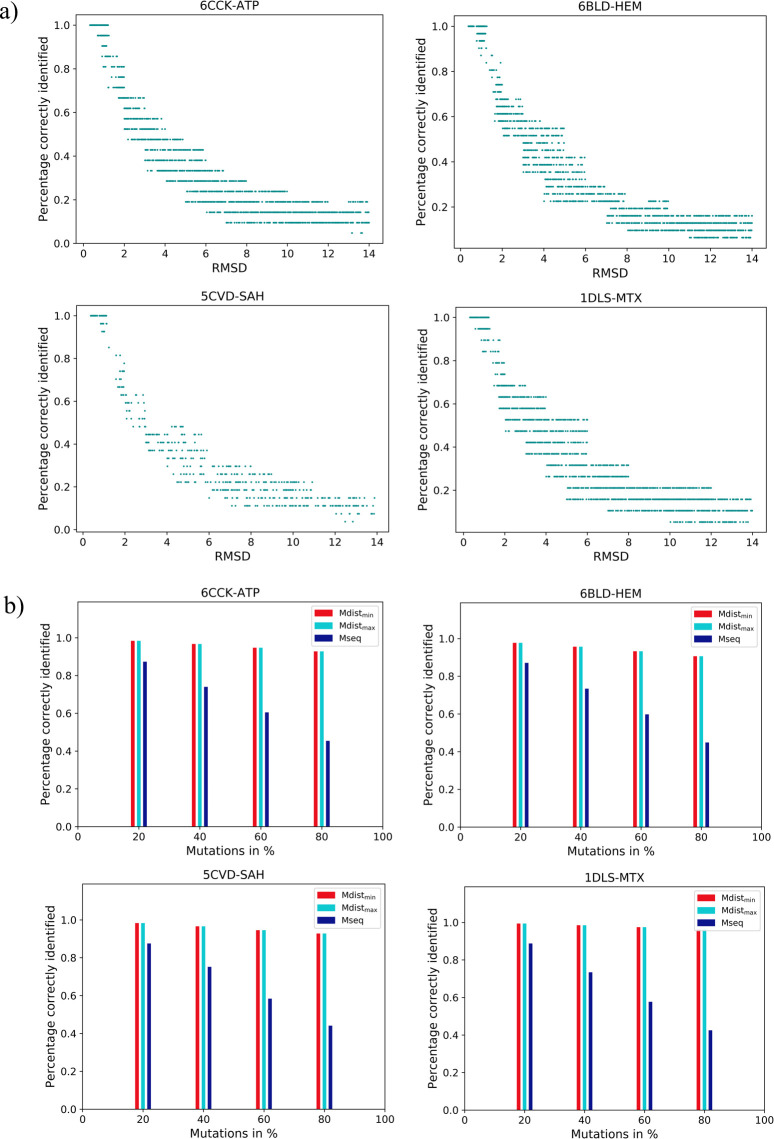
Sensitivity Analysis of SiteMotif: Performance of SiteMotif upon random perturbations (A) in the positions and (B) of residue types, of site residues of the selected examples—PDB-LigID: 6CCK-ATP, 6BLD-HEM, 5CVD-SAM and 1DLS-Methotrexate binding sites. The residue positions in the sites of all ligand binding sites were randomly perturbed in one or more positions such that the RMSD values ranged from 0 to 14Å. The ability of SiteMotif to correctly identify the alignments was evaluated. In (A) cases where the same residue types but different inter-residue distances as compared to the original site were screened whereas in (B) cases where two sites share similar inter-residue distances but different residue types are tested. In A), a gradual decrease in M-dist_min_ score is seen as the extent of residue perturbation increases. M-dist_min_ scores remain high (> 0.94) for all cases of perturbation with RMSD < 1.5Å (B) As expected, M-dist_min_ score remains the same over the entire range of perturbation, while M-seq score decreases as the rate of mutation increases.

To study the influence of perturbation on position, residues in the binding sites were disturbed in one or many locations such that RMSD values range between 0 to 14Å. The perturbations mimic minor variations in site positions that can be expected due to scenarios such as poor crystal density in the region, high flexibility, model errors in the given protein, conformational changes in the site upon ligand binding. The residues in the sites were perturbed in one or more of their residue positions about 3000 times for each pair such that the RMSD value with its original counterpart ranged from 0 to 14Å. We indeed observed that the perturbed sites were correctly aligned for all pairs when the RMSD was less than 1.5Å (Fig 2A). In all, we found 783 pairs in which the perturbation was within 1.5Å. The accuracy of alignment decreases gradually as the RMSD increases, illustrating the sensitivity of SiteMotif to distance perturbations. In all cases where the perturbations are below 1.5Å, the nature of binding sites within each group was observed not to be disturbed and SiteMotif identifies them correctly and performs robustly.

### b) Sensitivity with respect to random perturbation of residue types

Next, we tested the sensitivity of SiteMotif to variations in residue types while retaining the atomic positions, with an aim of mimicking scenarios of aligning pockets that are geometrically similar but exhibiting sequence variation. Towards this, we randomly mutated different percentages of residues to randomly selected residue types. The same dataset used in the previous section was used to generate 3000 random mutant pockets for each chosen example and measured the sensitivity of SiteMotif for the mutational perturbations. From Fig 2B, it was evident that even after mutating 80% of residues, the M-dist_max_ score remained as 1. This is because, in this perturbation set-up, although both wild-type and mutant binding pockets have different residue compositions, they share similar intra-residue distances, hence yielding high M-dist scores despite low M-seq scores in such synthetically perturbed pocket pairs.

For the remaining 20 ligands, we observed a similar pattern for both distance-based perturbation as well as mutational perturbations, further improving the effectiveness of our method in handling diverse scenarios (S5 File).

### Validation of SiteMotif

SiteMotif provides structural motifs for a given set of binding sites if they exhibit site-level similarities, irrespective of their sequence and fold-level similarities. In the process, it generates (a) pairwise structural alignments of a given pair of pockets and, more importantly, (b) a common aligned framework of multiple sites. We designed the validation protocols to test each of these aspects. Accuracy of SiteMotif was tested across five categories of protein sets as mentioned in Table 1. Level-1 constitutes those protein pairs that share high similarity at all levels i.e, similar sequence, similar structure, similar binding sites and recognizing the same ligand. To address this, we used a pdb_95 dataset downloaded from the PDB, where clusters formed by sets of proteins that share > 95% identity among them were identified [21]. The dataset pdb_95 constitutes 2800 groups, out of which we ran SiteMotif on 2254 clusters (32914 Pairs) and estimated residue conservation on all of them. For the remaining 546 clusters, the number of members was less than three and were therefore not considered. An all-vs-all comparison of sites within each cluster shows high conservation of residues with a mean RMSD of 0.4Å (S1 File—sheet1). The accuracy of residue-residue alignments of six randomly chosen clusters is shown in S3 Fig.

**Table 1:** Estimating the sensitivity of SiteMotif for proteins sharing similarity at each level. Level-1 constitutes those protein pairs that share high similarity at all levels i.e similar sequence, similar structure, similar binding sites and recognise the same ligand. Whereas for level-2, similarity is observed at all levels except at the sequence with an average sequence identity less than 30%. Level-3 constitutes testing the performance of our method from the known literature reports.

Levels	Level-1	Level-2	Level-3	Level-4	Level-5
Sequence	✔	✖	✖	✖	✖
Fold	✔	✔	✖	✖	✖
Site	✔	✔	✔	✖	✖
Ligand	✔	✔	✔	✔	✖
No. of PDBs	30405	48	46	27	28113
Remarks	True hit (Positive control)	SCOP Database	Known datasets from the literature	ATP (Negative control)	Random sets of unrelated proteins (Negative control)
SiteMotif results	SiteMotif outputs correct alignments in all cases	SiteMotif outputs correct alignments in all cases	SiteMotif outputs correct alignments in all cases	SiteMotif correctly identifies these as dis-similar; No alignment is obtained	SiteMotif correctly identifies these as dis-similar; No alignment is obtained

**Fig 3:**
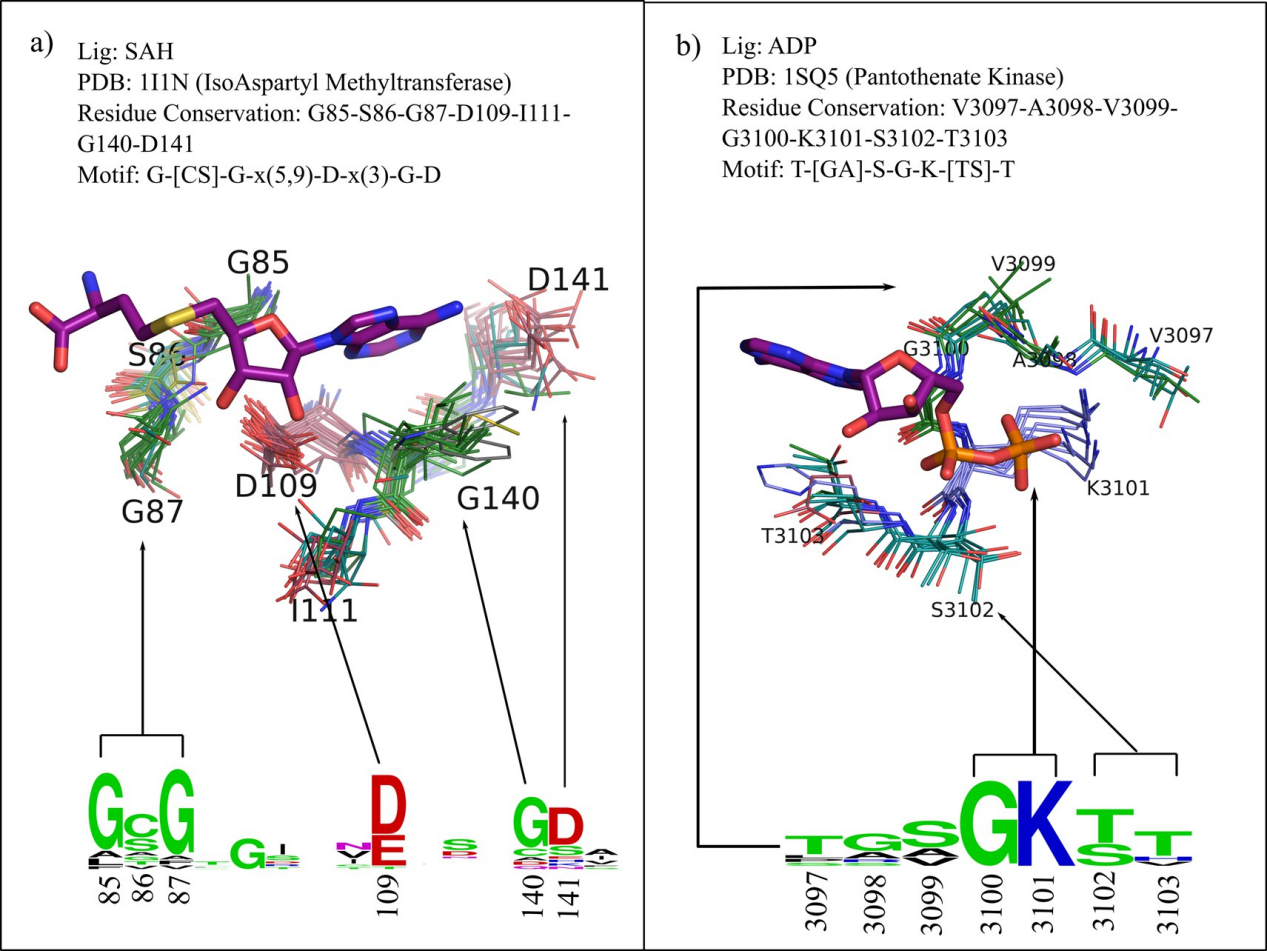
Alignment output from SiteMotif showing site conservation in the binding sites of proteins having no sequence relationships in 2 different cases. A) SAH binding proteins—7 site residues are very well aligned in all structures (31 structures belonging to 31 different families); sequence GSG was highly conserved near the methionine moiety of the ligand. Two negatively charged residues D109, D141 positioned appropriately to make strong hydrogen bonds with the SAH are observed. B) ADP binding proteins—Sites from 11 different proteins belonging to 11 different families show excellent alignment in 7 site residues. An amino acid segment TGSGK was observed to be a conserved structural motif. This includes a lysine (K3101), making hydrogen bonds with the β-phosphate of ADP.

For level-2, we analyzed a set of proteins from the SCOP database that recognize the same ligand and belong to the same structural superfamily but share no obvious sequence similarity [16]. For this study, we found 13 clusters and briefly analyzed two example ligands SAH and ATP that were known to bind to a wide array of protein families. SAH binding proteins of the c.66.1 SCOP superfamily belonged to 31 families whereas ADP binding proteins of c.37.1 superfamily belonged to 11 families [16]. Sites for each of the ligand binding proteins were compared in an all-vs-all manner. Representative sites for SAM/SAH and ATP/ADP were identified as described earlier. S2 Table provides the detailed description of the number of unique SCOP families used, sequence identity of each member with its representative and the calculated RMSD of site residues obtained from SiteMotif. IsoAspartyl methyltransferase, the selected representative, carries out a reversible conversion of isoaspartyl to aspartate during the process of protein repair [22]. SiteMotif aligned the sites well (Fig 3) with an average M-dist_min_ score of 0.57. Further, SiteMotif identified G-[CS]-G-x(5,9)-D-x(3)-G-D as the motif. One aspartic acid was seen to be appropriately positioned to make a hydrogen bonding with ribose oxygen, where another aspartate interacted with the amine group in the adenine ring of S-Adenosyl-l-homocysteine (SAH) ligand. The residue sequence GSG that was found to predominate near the methionine is known to be highly conserved in proteins binding to SAM/SAH. Similarly, for the c.37.1 ADP superfamily, Pantothenate Kinase was the representative, which catalyzes the conversion of pantothenate to 4’-phosphopantothenate using ATP as a phosphate donor [23]. SiteMotif aligned the sites well with an average M-dist_min_ of 0.68 and identified the motif T-[GA]-S-G-K-[TS]-T, which is well characterized as the Walker motif associated with nucleotide binding [24]. Level-1 and Level-2 validation serve as positive controls. Detailed information about the PDB identifiers, SCOP identifiers, and ligand identifications of all 13 clusters is provided in the supplementary file (S1 File—sheet2).

**Fig 4:**
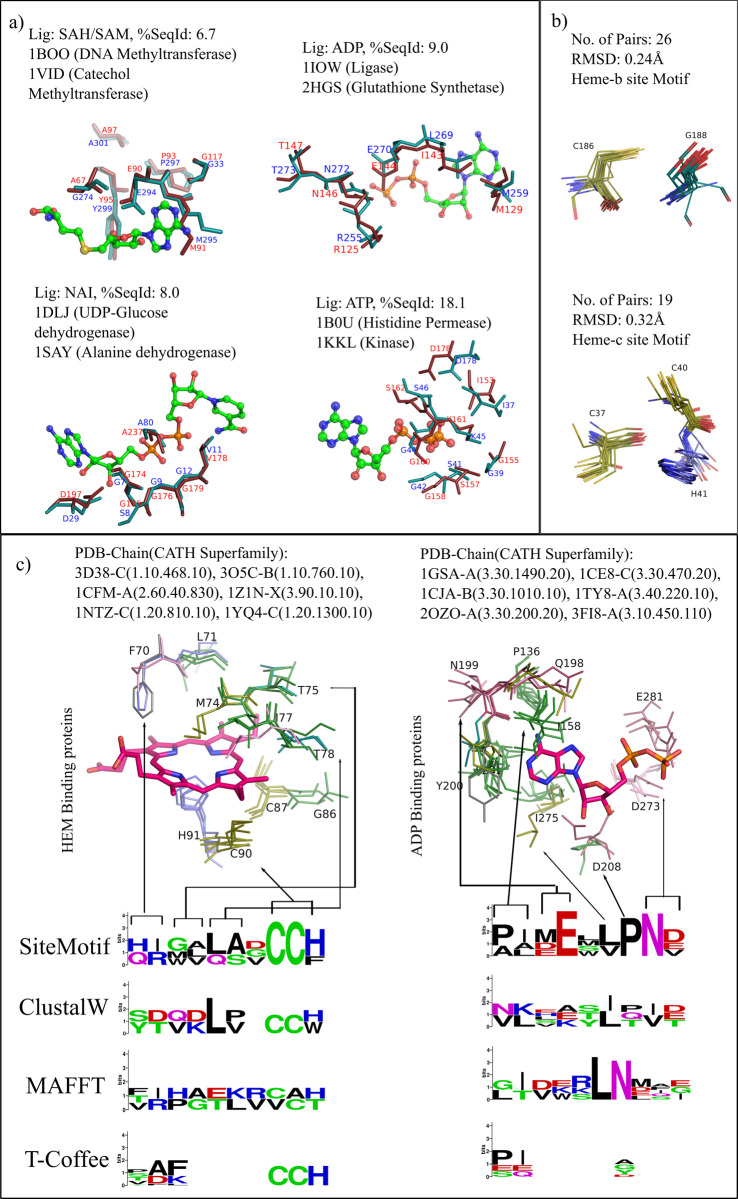
SiteMotif output of Site similarity in the pockets of proteins belonging to diverse folds. A) Pairwise comparison was carried out for four pairs of proteins that recognize the same ligand but have very low sequence similarities (value of percentage sequence identity (%SeqId) is shown for each pair). B) Test for accuracy of multiple alignments using two protein sets, one complexed with heme b ligand and the other complexed with heme c. The motif CXG was identified to be conserved for heme b binding proteins, whereas motif CXXCH was conserved in the sites of protein interaction with heme type c. The average structural deviations of the aligned residues were less than 0.2Å. In all cases, conservations reported by SiteMotif match well with the original study from which these datasets were obtained. C) Showcasing the importance of site alignment over sequence alignment tools in finding conservation across ligands binding to different CATH superfamily.

Level-3 involves testing of protein-pairs with no sequence or fold similarity but with an apparent site-level similarity that can recognize the same ligand. Level-4, on the other hand, involves the study of protein pairs with no sequence, fold, or site-level similarities but recognizing the same ligand. Levels 3 and 4 were more challenging and required the use of site substructure comparison methods as they cannot be inferred through sequence or fold level methods. There are not many methods to achieve this, but there are many individual examples from literature where similarities were studied in selected proteins. We performed level 3 using three different datasets of similar sites from dissimilar structures, reported previously, The first dataset was obtained from PLIC (Protein Ligand Interaction Cluster) database which is based on an exhaustive comparison of all pairs of 84,846 known ligand binding sites from PDB [25]. All pairs that exhibited similarity (PocketMatch Pmax > 0.6) were fed into Markov clustering (MCL) algorithm to obtain binding site clusters. Of 10,858 clusters reported in PLIC, nine clusters constitute sites from proteins belonging to different CATH families and binding to similar ligand compounds, which are taken for analysis here. PLIC entries are annotated with CATH superfamily, GO terms, EC number, ligand binding strength and atomic contacts between protein and ligand. We analysed the top two predominant PLIC clusters (cluster IDs 1 and 3) and tested if the motifs obtained from SiteMotif could be inferred solely from the primary sequence taken from the multiple sequence alignment using three popular programmes ClustalW, MAFFT and T-Coffee. Cluster 1 comprises seven binding site entries where each protein belongs to distinct sequence families (average sequence similarity among the 6 was < 30%) and distinct CATH families (CATH Id: 1.10.468.10, 1.10.760.10, 2.60.40.830, 3.90.10.10, 1.20.810.10 and 1.20.1300.10) but bind Heme or its structurally similar ligands (HEC). Fig 4C represents aligned regions present in the sites of six heme binding CATH families along with weblogos obtained using two different approaches (SiteMotif and ClustalW/MAFFT/T-Coffee). The known motif ‘CCH’ was more prominent from the result of the SiteMotif than that obtained purely from the multiple sequence alignment. In fact, all three sequence alignment methods failed to pick the presence of CCH in a few proteins (PDB ID: 1CFM, 3O5C) that possess the desired motif. Similar trend was seen for the entries present in PLIC Cluster 3 too, where the majority of proteins possess ADP ligands in their binding sites and share no similarities at sequence and fold. Six proteins, one for each CATH family, were taken to compare the conservation of motifs obtained from SiteMotif and sequence alignment methods. SiteMotif correctly identifies known conservatives such as aspartate and asparagine whose importance towards ligand binding was previously well characterised [26].

The second dataset for this study was obtained from Ausiello et al [27] where cases of possible convergent evolution were described, identified by the authors by the following approach: From PDB, a total of 1924 non-redundant protein chains were retrieved from which 2500 functional binding sites were predicted based on known ligands or PROSITE patterns. Using an all-vs-all site comparison by Query3d [28], 4 different pairs were identified that were not related by sequence but had similar binding sites. We applied our method onto these pairs and observed that all residues that were identified in Ausiello et al study were correctly picked by SiteMotif (Fig 4A). The third dataset was from another study that described new motifs in the Heme binding site from a set of non-redundant proteins from PDB [29]. Here, the authors carried out two independent site comparison studies one for heme-b ligand from 26 proteins and the second for heme-c ligand from 11 proteins. Similarities in the binding sites were measured based on the ratio of the volume and the surface area of each pocket. Two motifs, one for each of the heme ligands, were identified. The motif CXXCH was found to be present in most heme-c binding proteins and CXG was seen in the sites of proteins binding to heme-b. We checked if SiteMotif could find these patterns. We carried out an all-vs-all site comparison for heme-b recognising proteins, which amounted to 676 pairwise combinations from which one representative, Cyanobacterial Cytochrome P450, was selected. The selection was based on a projection graph as described earlier, where sites were the nodes and the extent of similarity between each pair was taken as the edge weight and the highest weighted degree node was identified as the representative. A similar analysis was carried out for the heme-c set as well. SiteMotif indeed yielded the expected alignments and correctly identified the motifs CXG in the heme-b set and CXXCH for the heme-c set (Fig 4B).

For level-4, diverse proteins binding to the same ligand but belonging to different sequence and fold families and having no obvious binding site similarities were tested. Getting datasets of this category is difficult as we have very few examples from the literature that report recognition of the same ligand by diverse binding sites. Recently, we reported a study on diverse nucleoside triphosphate (NTP) binding sites in which we developed a non-redundant set of 27 protein representatives from PDB representing different NTP site-types [30]. The only common thing among them was their ability to bind to NTPs. As expected, SiteMotif outputs no similarities between them indicating the sensitivity of our algorithm. The final category, Level-5, involves a pool of random sites with different residue composition, different geometry and binding to different ligands. More than 20,000 of such pairs were studied and no similarities were detected in any of them by SiteMotif, again serving to demonstrate the sensitivity of the algorithm. Levels-4 and 5 serve as negative controls.

### Benchmarking SiteMotif against other binding-site comparison algorithms

**Fig 5:**
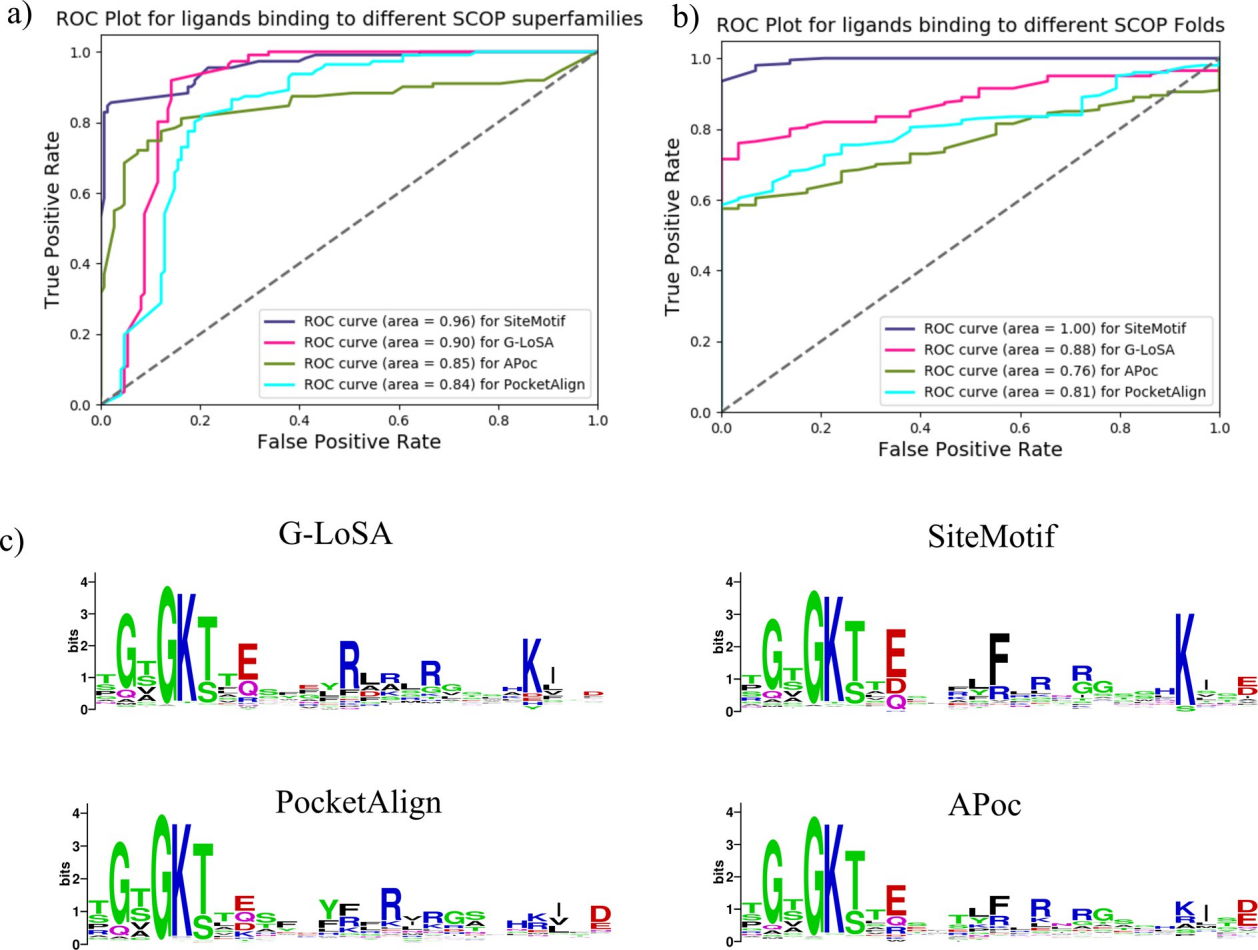
ROC curves of SiteMotif against widely used site alignment programmes G-LoSA, APoc and PocketAlign. The similarity score was computed by dividing the number of residues aligned by the number of residues of the smallest binding sites. A) Includes pairs of binding sites from proteins of different families but of the same SCOP fold and known to bind the same ligand. All 4 algorithms detect similarities well and the length of the alignment does not vary much. B) Includes pairs of binding sites from proteins adopting different folds but sharing structural similarity only in the local region of the binding sites. SiteMotif performs better than other methods and identifies distantly gapped structural motifs that were not detected by other methods. G-LoSA came out as the second best performing tool. C) Weblogo showing the conservation of ATP binding sites derived using different site alignment programs.

The capability of SiteMotif can be dissected into the following components: (a) pairwise site alignment, (b) placing them into a common structural framework and (c) subsequently deriving motifs. Methods capable of identifying automatic detection of structural motifs from large inputs of sites are currently not well established. However, a number of methods have been developed for pairwise comparison. To test how SiteMotif fares with respect to these in terms of achieving pairwise site structure alignment, we measured the sensitivity of our method against three state-of-the-art site alignment methods G-LoSA, APoc and PocketAlign. APoc (Alignment of Pockets) tries to find optimal alignment between two protein binding sites which involves generating a series of gapless alignments of local contact patterns, iterative dynamic programming to get optimal alignments of pockets using a linear sum assignment problem [7]. Glosa (Graph-based Local Structure Alignment), on the other hand, constructs product graphs for input binding sites using cα-alpha distances and finds the most significant subset of vertices using a branch and bound algorithms followed by iterative maximum clique search algorithm to find the fragments which are then superposed using a rotation matrix [8]. PocketAlign obtains structural superpositions by computing geometric perspectives and captures the relative distribution of residues around each residue in a given site, supplemented by chemical descriptors. The perspectives are then compared between a pair of sites initially through seed alignments which are subsequently extended to get maximal consequential atomic alignments [9]. We performed this analysis on two categories of proteins at different levels of anticipated difficulty in identifying similarities. (i) First, we took 111 sets, where each set contains sites that bind to the same ligand from the same fold but belong to different SCOP families. As proteins in each set share high structural similarity, the site-level similarity was expected to be easily identified. All three methods performed well for all pairs in obtaining pairwise alignments (Cɑ -Cɑ distances of corresponding residues < 1.25 Å). However, for some pairs, differences in the number of residues matched by three methods vary by more than 5 residues (S4 File). SiteMotif was seen to have the best performance in identifying the maximum number of aligned residues (Fig 5A). (ii) The second category of sites consisted of hard targets as we anticipated that similarities among them would be non-obvious. For this, we took 199 sets consisting of binding sites that recognise the same ligand but belonging to different SCOP folds. SiteMotif was seen to be the best performer among all methods studied here in finding the largest number of aligned residues and subsequently leading to distantly gapped sequence motifs (Fig 5B). G-LoSA identified such motifs for some pairs of binding sites but its performance was variable across proteins. APoc, which performs gapless alignments and secondary structure information for generating initial seeds, requires a minimum of 9 residues to report alignments, and was not best suited for this problem as similarities were found only in 14 of the 111 pairs and hence was not considered for comparison. The sample alignments produced by all four methods are shown in S4 Fig and full details on the list of site pairs, their SCOP hierarchy and alignments from different methods are provided in S4 File.

Our next goal was to test how our method fares in its second step, which is placing site-pairs onto a comparable framework producing an approximate multiple site alignment. None of the methods are designed to perform multiple site alignments or place site-pairs in a common framework, but it was possible to design a work-around by performing all-pair alignments with each method, selecting a representative and using it to place them obtaining a multiple site alignment. We used a set of non-redundant ATP/ADP binding proteins and asked how the four methods perform in obtaining the multiple site alignment. ATP/ADP binding proteins have been well characterized biochemically with a sound understanding of the contribution of individual residues and provide several examples for performing this type of a test. Specifically the Walker motif GxxxxGKS/T which is known to be conserved in sites of many proteins recognising ATP [24]. Following the high predominance of positively charged residues near the phosphates, a conservation of glutamic acid for the hydrolysis of ATP is also well documented. Hence we chose this example as a case to measure the accuracy of different methods towards motif derivation. From PDB, we retrieved a total of 1319 non redundant proteins that are complexed either with ATP or ADP ligands. Binding site residues are defined based on the distance threshold of 4.5Å from the ligand atom. An all-vs-all comparison resulted in 1,739,761 pairs. To avoid the run time complexity imposed by other methods, a representative site was selected (as described in Methods) against which all other sites were compared. Finally, the conservation of site residues reported by each method were inspected graphically using WebLogo. KaiC, a circadian clock protein (3K0C), that shares highest similarity with most members of the set, was taken as the representative. All other sites were aligned on to this independently using SiteMotif, G-LosA, APoc and PocketAlign. SiteMotif completed scanning 1,739,761 pairs in 68 minutes on 216 cores on a Cray supercomputer (~646 pairs per second). From Fig 5C, it is evident that the conservation output obtained from all the methods tend to be similar, but SiteMotif was much more efficient. Details about the PDB ID, binding site residues and residue matches reported by each alignment method are provided in a supplementary file (S2 File).

### Use cases

A tutorial describing the use of SiteMotif is provided in Supplementary File (‘Tutorial.pdf’). We envisage a number of scenarios where SiteMotif can be applied, such as improving protein function annotation and drug discovery. Some example putative use cases in the broad space of function annotation are (a) identification of conserved residues in proteins of different SCOP folds and superfamilies, (b) identification of critical residues that define ligand binding in any given set of proteins (ranking among binding site residues based on the extent of conservation), (c) designing loss of function and gain of function mutations and (d) detection of site-based sequence motifs and associate them with recognition of specific ligands. Many use cases can be thought of in the space of drug discovery also, which include optimising for specificity and selectivity during lead identification and optimization, drug repurposing and enhancing safety profiles by identifying and reducing off-target binding.

### New insights from SiteMotif: A case study that derives new structural motifs

**Fig 6:**
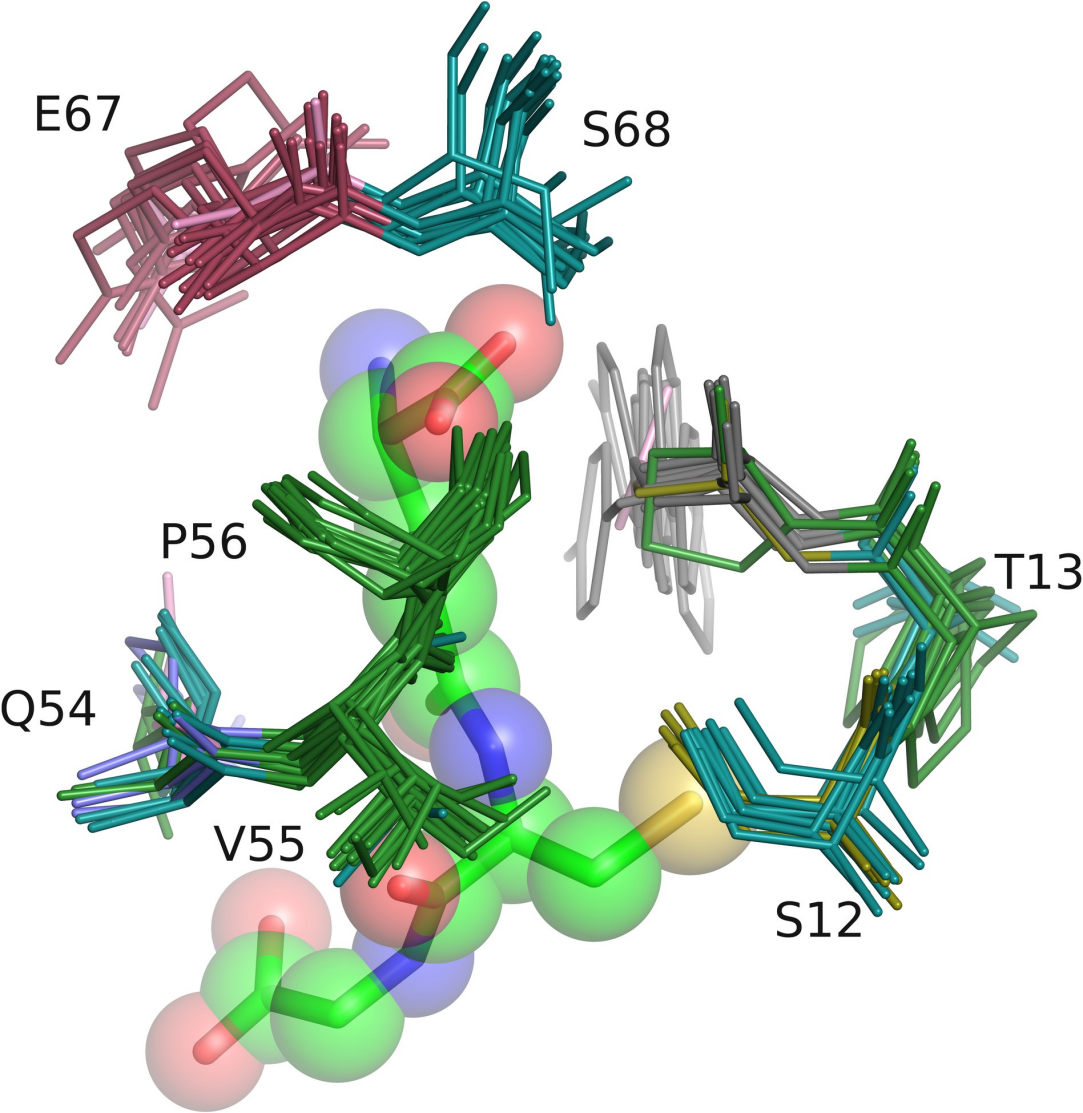
Showcasing the capabilities of SiteMotif in finding complex motifs that are not reported earlier for an example of glutathione binding proteins. Some of the residues have individually been studied and known to interact with the ligand but a motif is not known. SiteMotif identifies two motifs [CS]-P-[FNWY] and [KQRTN]-[IV]-P-X(9,25)-[QED]-S that are known to be predominant in proteins binding GSH (Glutathione).

We showcase the capability of SiteMotif in deriving motifs with a case study of glutathione binding proteins. A few residues that play a role in glutathione recognition have been identified through biochemical studies in a few individual proteins, but a motif has not been reported so far [31]. Glutathione (GSH) is a tripeptide made up of cysteine, glycine, and glutamic acid and plays a key role in maintaining the redox state of a cell. 336 proteins bound to glutathione were found in PDB, which when pruned at 30% sequence identity, resulted in a list of 15 non-redundant proteins. We analysed these using SiteMotif and derived a structure-based sequence motif of the glutathione binding site (Fig 6). These proteins which belong to the families of Glutaredoxins, Glutathione S-transferase and Glutathione S-transferase theta-1 adopt the same fold but do not share sequence similarity and have considerable differences in the residues at their binding sites. SiteMotif identified a 3D motif with two sequentially discontinuous parts [CS]-P-[FNWY] and [KQRTN]-[IV]-P-X(9,25)-[QED]-S, but spatially in the same vicinity, present at the GSH binding sites (Fig 6). SiteMotif was able to identify key residues at the site and tease out structurally conserved motifs critical for glutathione binding, from large binding sites. Biochemical evidence from literature suggests that the motif residues cysteine and tyrosine are responsible for catalysing glutathionylation reactions in glutathione s-transferases [32]. The residue proline from the motif has been known to be important for maintaining the structural integrity of glutathione s-transferases [33,34].

In order to test the sensitivity of the derived motif, we scanned the motif against the set of 336 known GSH binding proteins from PDB and were able to correctly identify all 336 GSH binding sites (average RMSD 0.4Å for all 8 aligned residues in the motif). Next, we carried out an unbiased scan for the GSH motif against all small-molecule binding pockets from PDB, using SiteMotif. For this, we used PocketDB, a recently reported resource from our laboratory [13]. PocketDB contains 113,486 known sites corresponding to all small molecule binding sites (filtered at 70% sequence redundancy). Using SiteMotif, the derived glutathione motif was scanned against the entire PocketDB site repository. In addition to the known GSH binding sites, 3 new sites that contained the motif were identified. These are (i) the bacteriocin transport accessory protein (PDB: 1ZMA), (ii) dihydrolipoamide dehydrogenase (1OJT) and (iii) Thioltransferase (1KTE). Dihydrolipoamide dehydrogenase (1OJT), is a flavoprotein responsible for oxidizing dihydrolipoamide, and adopts the glutathione reductase fold [35]. CATH analysis of the Bacteriocin transport accessory protein indicates that it adopts the glutaredoxin family (3.40.30.10) fold [36]. This protein from *S*.*mutans* was shown in a proteomic screen to undergo S-glutathionylation by binding to glutathione in its active site [37]. A residue from our GSH motif (C^38^PYC^41^) present in this protein was shown to be crucial for glutathionylation [37]. For the third, although there was no reported evidence linking Thioltransferases (1KTE) to GSH binding, we used FunFams, a bioinformatics tool that predicts function based on sequence analysis, which classified Thioltransferases as glutaredoxins, which are known GSH binding proteins [38]. Molecular docking was carried out to estimate the theoretical feasibility of binding by calculating intermolecular energies of the identified hits with glutathione, which showed favourable binding energies in all three cases (S5 Fig and S3 File).

## Discussion

Structural bioinformatics is an area that rationalizes and classifies information in three dimensional structures of protein molecules and facilitates mining complex patterns that provide insight into functional capabilities of proteins and ultimately aid in understanding biological processes at atomic level detail. Given the large number of structures that are accumulating in PDB, it is becoming important to understand structural motifs and specific residue associations with the functions of the respective proteins. This emphasizes the need for designing appropriate tools to be scalable and fast without compromising the accuracy in getting the final alignment. The importance of multiple alignments as a concept needs no special persuasion as multiple sequence alignments have found extensive application in modern biology. A number of algorithms and web-accessible software tools have been developed for this purpose, each employing a distinct approach to optimise the objective of obtaining high scoring alignments, common examples being CLUSTAL-W, T-Coffee and MUSCLE [39–41]. A similar tool but at the level of three dimensional structures will offer deep insights into structure-function relationships. The Program SiteMotif was developed to meet this requirement. The algorithmic design of SiteMotif implements the concept of graph traversal by representing residues as nodes and distances between them as edges. Three types of edge distances were computed using c⍺, cβ and cn points, enabling accurate and faster match operations. The final output of SiteMotif includes three scores catering to different needs, and also the list of matching residues in each site, as well as the final multiple alignment. Construction of the multiple alignment starts with an identification of correspondences at the residue level for all combinations of site pairs from which a conservation profile is generated. These structure based sequence profiles serve as a basis for deriving the motif.

An obvious question that comes to mind when a new algorithm is developed is that—where does it stand in the context of present day advances in the field? Many binding site comparison tools have indeed been developed over the last decade, which can be grouped into ‘Alignment Based’ (eg- PocketAlign, G-LoSa and Probis) or faster but less sensitive ‘Alignment free’ methods (eg., PocketMatch and RAPMAD). The latter methods are fast and hence suitable for large scale comparisons. Nevertheless, they report only the similarity score and not residue correspondences, which alignment based methods provide. Contrary to sequence and structure, alignment of binding sites poses a number of challenges as they form discontinuous point sets and show inherent sequence order independence, both of which leading to a very large search space. Existing pairwise methods can still be tweaked to get such alignments but they often require additional parameters such as structure of the whole fold or presence of ligand in the site along with the binding site coordinates. Moreover, alignment based operations tend to perform slower due to the inherent nature of sequence order disconnectivity in the binding sites. Not all methods are fast and parallelizable, restricting themselves to a maximum of a few thousand operations. Our program SiteMotif is designed to work seemingly on both regular desktop machines as well as to harness the power of high-performance supercomputing clusters and it does not require the knowledge of the protein fold for doing the comparisons.

Site motifs in general, serve as the robust recognition parameters for a protein to interact with small molecules (eg: Walker motif- ATP binding). Precise identification of site motifs is therefore expected to be very useful for understanding determinants of function. Structure-based annotation tools stand out as reliable methods to derive functional insights from orphan proteins or proteins of unknown function. The inclusion of binding site similarity as an additional feature is expected to enhance the accuracy and the case coverage of such operations. Several studies have shown that there are many cases where different proteins can bind the same ligand, indicating the acute need for a tool to enable their rationalization. Comparing sites and identifying key determinants of ligand recognition is important in drug discovery applications as well. The need for studying the characteristics and conservation between binding sites stems largely due to identification of off-targets causing an undesired physiological response upon various drug treatments. Increasingly, such off-targets are being reported for many drugs. Identification of motifs can facilitate an understanding of which interactions with the protein are dispensable and which are critical, providing a basis for designing drugs with higher specificity and selectivity.

In conclusion, we present a new robust sensitive algorithm for deriving structural motifs in proteins. SiteMotif can detect motifs even in cases where proteins bind the same ligand but share no sequence or fold level similarity. We expect that this tool will find use in a number of basic science as well as application projects.

## Supporting information

S1 Fig A bar plot representing the performance of SiteMotif in finding correct sites at different M-dist_max_ thresholds. The dataset reported in S1 Table has been used. The sites are compared in an all-vs-all fashion for each SCOP superfamily, and the final output is analysed at the chosen M-dist_max_ threshold (0.4, 0.5, 0.6, 0.7, and 0.8). At M-dist_max_ > 0.4, the binding sites of all proteins align well with each other.
(TIF)

S2 Fig Run time performance and scalability of SiteMotif across different numbers of processors on a distributed cluster. A random set of 10,000 pairs of ATP binding sites was used to test our program’s scalability in the Cray supercomputer. As seen from the graph, the execution time of SiteMotif scales well with the number of CPU cores. Such parallelization enables faster and accurate detection of residue correspondence of millions of site pairs in a day.
(TIF)

S3 Fig Evaluating the alignment accuracy of SiteMotif in the binding site of proteins present in the level-1 category. A set of six clusters, each recognising distinct ligands were selected randomly from the pdb_95 dataset—PDB-LigID: 3JQG -NAP, 6GX2 -UDP, 1OTB -HC4, 4E1G -NAG, 4F2V-CXM, 6R49ADP. Representative protein for each member was selected based on M-dist_max_ > 0.6, upon which the least-squares structural superposition was carried out against all members of a site using the Kabsch algorithm. In all cases, SiteMotif successfully identified all site residues to be conserved.
(TIF)

S4 Fig Alignments obtained for the same pair of sites using all four methods; Two different cases are shown. Site alignment of each pair indicating the number of aligned site residues in case of (A) proteins of the same fold but different SCOP families—Guanidinoacetate methyltransferase (c.66.1.16, PDB:3ORH, Lig:SAH) and Glycine N-methyltransferase (c.66.1.43, PDB:1WZN, Lig:SAH). B) diverse proteins binding with the same ligand—Heme-dependent peroxidases (a.93, PDB:1BGP, Lig:HEM) and Globin-like (a.1, PDB:3ZHW, Lig:HEM). Here similarity is not detectable both at the sequence and the structure, highlighting the use of the method to find commonalities in the site of these proteins. In both the cases, SiteMotif reported the highest number of aligned residues, which was followed by G-LoSA and then by Pocketalign and APoc.
(TIF)

S5 Fig Docked poses of glutathione along with the interaction energy in newly identified proteins. From PocketDB, ligand binding pockets of all proteins are taken and compared with the derived motif for glutathione. Residues present in the binding site in each case are also shown. Glutathione motif residues are shown as thicker lines, while glutathione is in ball-and-stick representation. The software AutoDock was used to calculate theoretical binding affinity between protein and ligand.
(TIF)

S6 Fig Scatter plot illustrating the distribution of ligand molecules present in the PDB. For every ligand, we derived two chemical descriptors: 1) the molecular weight and 2) the partition coefficient. LogP is a direct correlation between ligands and solubility. The higher the LogP the more lipophilic the ligand. From the descriptors, a spaced 2D bin was created, from which 20 distinct ligands, one from each grid, were chosen for sensitivity testing.
(TIF)

S1 Table Assessing the threshold cutoff of M-dist score and the quality of final site alignment associated with three different SCOP superfamilies. Superfamily c.37.1 constitutes a total of 15,129 pairs of sites binding to adenosine diphosphate. Similarly, proteins belonging to c.23.5 (FAD binding proteins) possess 3136 pairs of sites, and a.1.1 (Heme binding proteins) comprises 345 site pairs. The binding sites of each of three SCOP superfamilies were taken to test the sensitivity of SiteMotif scores. MPI version of SiteMotif was used to compare 65,789 site pairs which completed the job in 3 hours on an 8 core Linux architecture. For each entry, the conservation index for each residue was computed as the fraction of the number of residues aligned by the total number of residues present. The final column represents the output of site alignment obtained using SiteMotif along with the sequence logo generated using WebLogo.
(DOCX)

S2 Table Testing the performance of SiteMotif on protein binding to the same ligand and adopts the same fold but unrelated at the sequence (level-3 dataset). Two predominant SCOP superfamily well reported to recognise cognate ligands was used here. Superfamily S-Adenosyl-L-Methionine-dependent methyltransferases (c.66.1) majorly binds with s-adenosyl methionine (SAM) ligand while P-loop containing nucleoside triphosphate hydrolase (c.37.1) binds to adenosine diphosphate (ADP) molecule. Both SAM binding protein and ADP binding protein sites align well in all structures with an average RMSD of 0.53Å and 0.52Å, respectively.
(DOCX)

S1 File Level Datasets–Contains detailed information about list of protein used to validate accuracy of SiteMotif across multiple levels of diverse scenarios (see Box 1). (XLSX)

S2 File Multiple Methods–Contains information about of list of proteins, used to measure the performance of SiteMotif in generating the sequence conservation of ATP sites against widely adopted site alignment programs, G-LoSA, PocketAlign and APoc. (CSV)

S3 File Pocketome-GSH–PDB ID’s of Glutathione binding proteins in PDB. (XLSX)

S4 File ROC-Datas–Measuring the sensitivity of SiteMotif against existing site alignment tools. Two independent exercises were carried out to test the alignment capabilities. A) Easy target—Comprises a list of proteins originating from the same SCOP folds and recognizes the same ligand but belongs to a different family. The compared proteins share a pretty good similarity in the binding site and hence have to be picked correctly. B) Hard target—Constitute pairs of protein that binding with the same ligand but are placed in different SCOP folds. As such that they don’t share any structural similarity between them.
(XLSX)

S5 File SensitivityAnalysis–Result of Sensitivity analysis of remaining 20 ligands (section ‘Sensitivity Analysis’). (XLSX)

S6 File Tutorial–A Tutorial describing the usage of SiteMotif for both pair-wise and multiple-site alignments. (PDF)
