# Supplementary figures and images for: SiteMotif: A graph-based algorithm for deriving structural motifs in Protein Ligand binding sites
Source: PLoS Comput Biol. 2022 Feb 24;18(2):e1009901. doi: 10.1371/journal.pcbi.1009901 (PMC8903255; doi:10.1371/journal.pcbi.1009901)

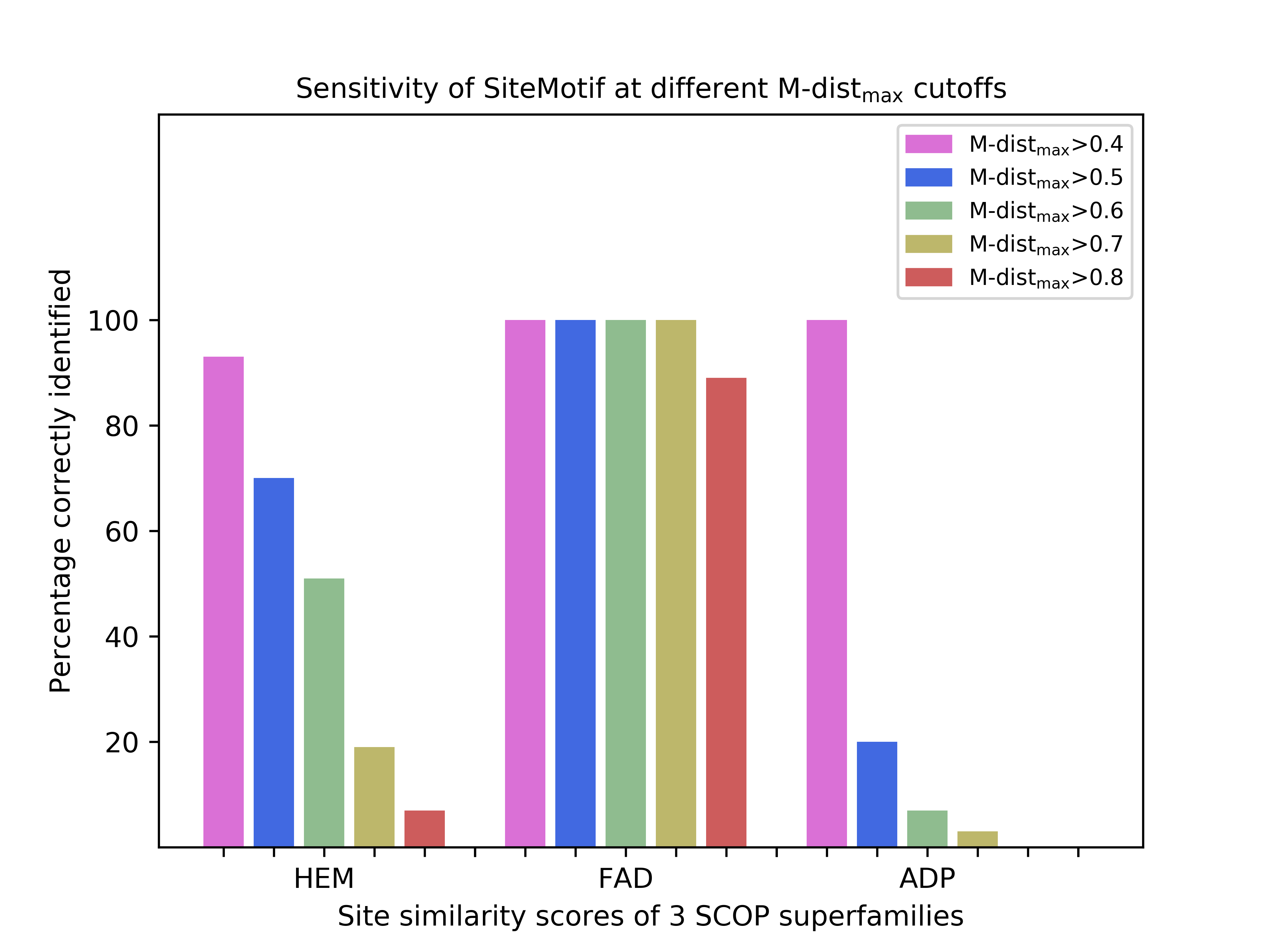

Supplement: S1 Fig — The dataset reported in S1 Table has been used. The sites are compared in an all-vs-all fashion for each SCOP superfamily, and the final output is analysed at the chosen M-distmax threshold (0.4, 0.5, 0.6, 0.7, and 0.8). At M-distmax > 0.4, the binding sites of all proteins align well with each other. (TIF) [file pcbi.1009901.s001.tif]

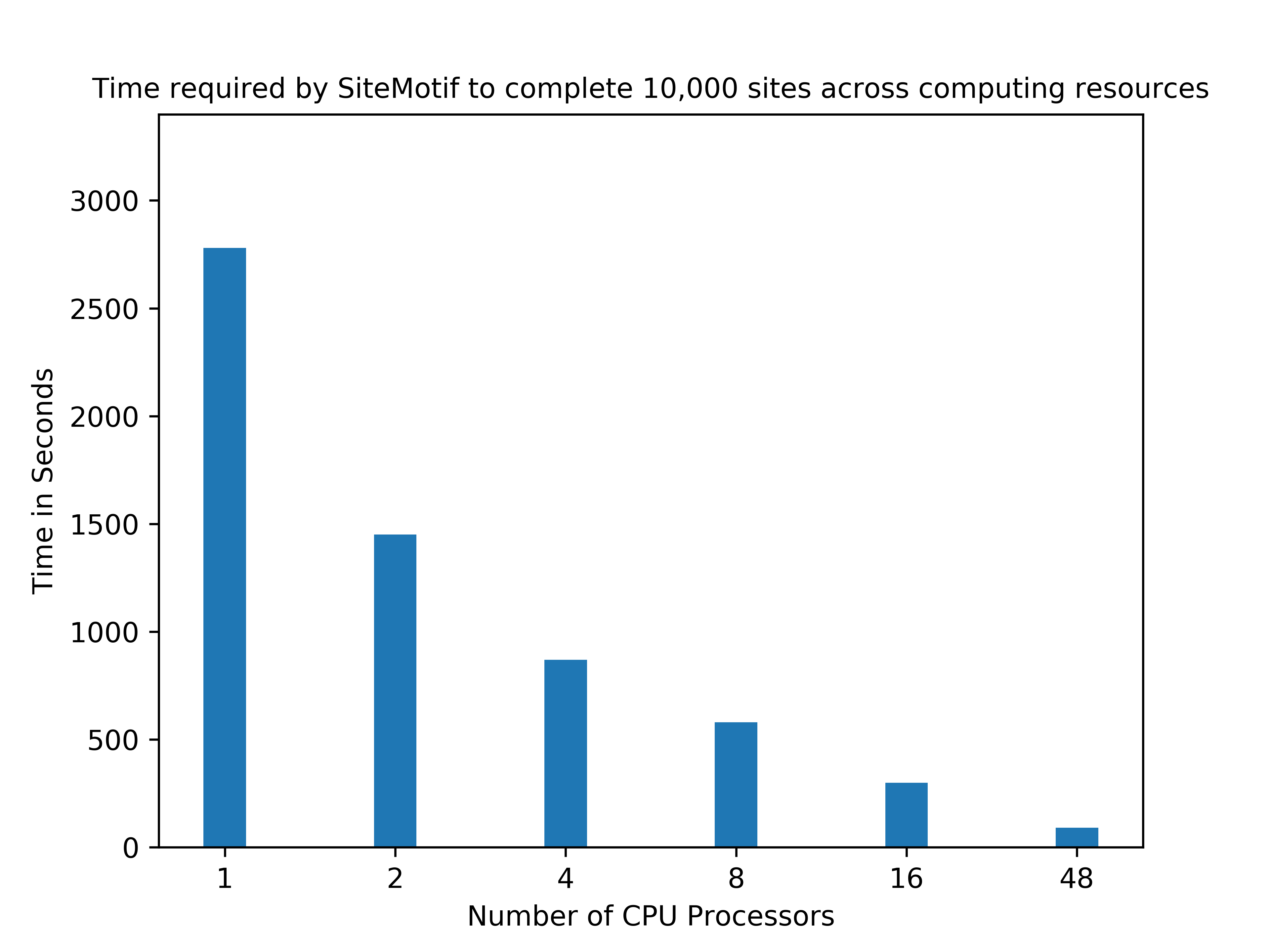

Supplement: S2 Fig — A random set of 10,000 pairs of ATP binding sites was used to test our program’s scalability in the Cray supercomputer. As seen from the graph, the execution time of SiteMotif scales well with the number of CPU cores. Such parallelization enables faster and accurate detection of residue correspondence of millions of site pairs in a day. (TIF) [file pcbi.1009901.s002.tif]

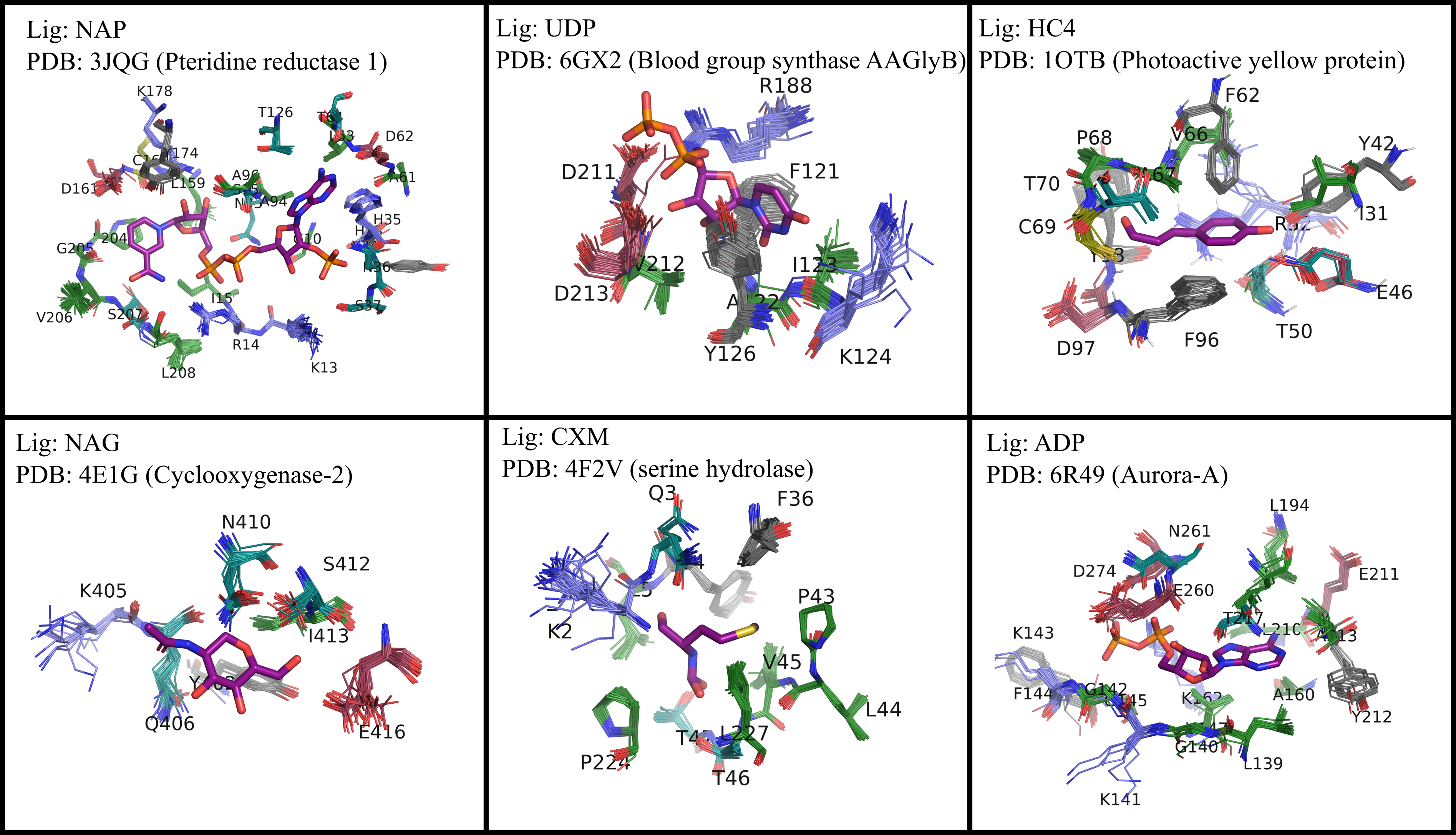

Supplement: S3 Fig — A set of six clusters, each recognising distinct ligands were selected randomly from the pdb_95 dataset—PDB-LigID: 3JQG -NAP, 6GX2 -UDP, 1OTB -HC4, 4E1G -NAG, 4F2V-CXM, 6R49ADP. Representative protein for each member was selected based on M-distmax > 0.6, upon which the least-squares structural superposition was carried out against all members of a site using the Kabsch algorithm. In all cases, SiteMotif successfully identified all site residues to be conserved. (TIF) [file pcbi.1009901.s003.tif]

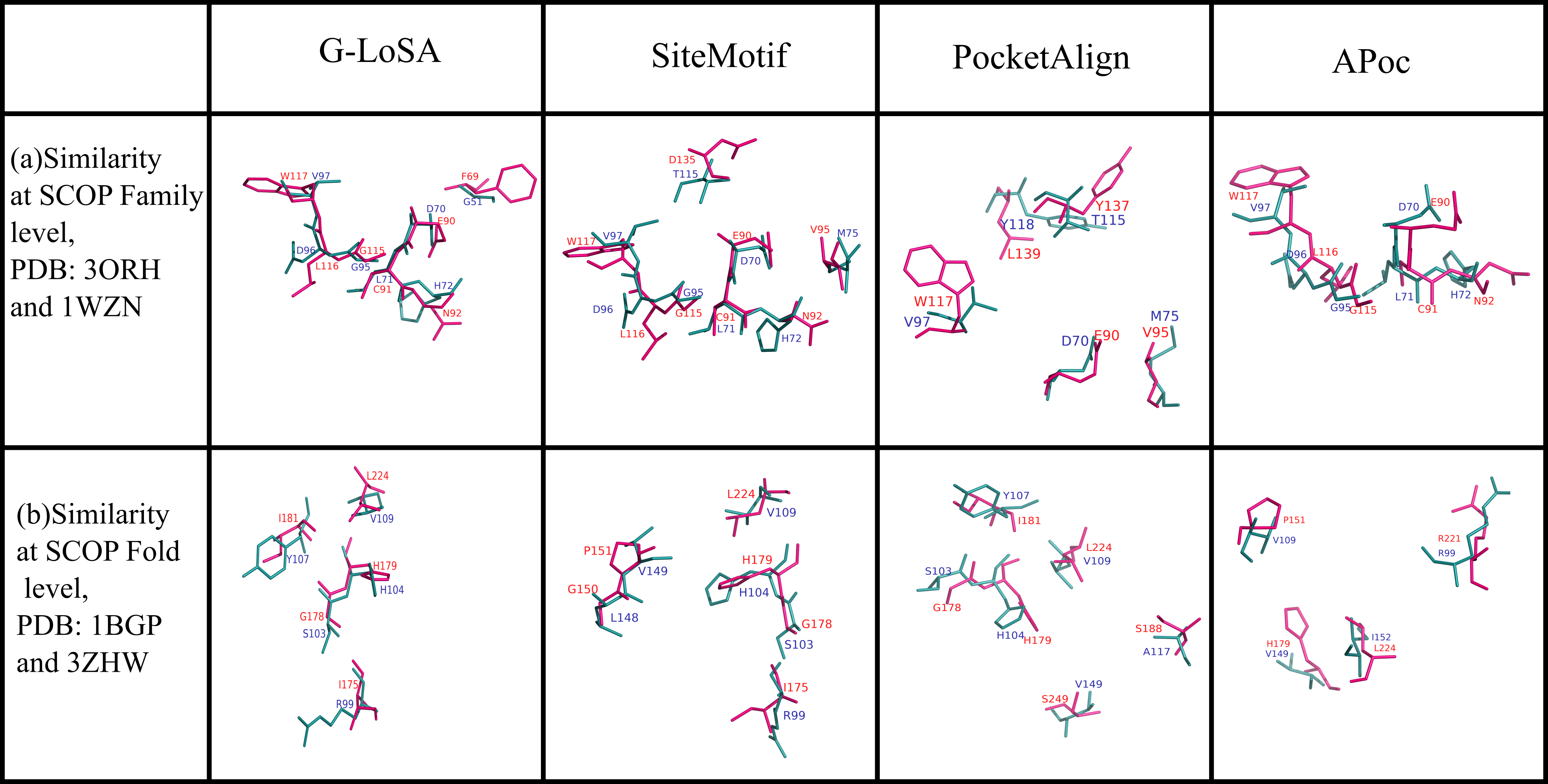

Supplement: S4 Fig — Site alignment of each pair indicating the number of aligned site residues in case of (A) proteins of the same fold but different SCOP families—Guanidinoacetate methyltransferase (c.66.1.16, PDB:3ORH, Lig:SAH) and Glycine N-methyltransferase (c.66.1.43, PDB:1WZN, Lig:SAH). B) diverse proteins binding with the same ligand—Heme-dependent peroxidases (a.93, PDB:1BGP, Lig:HEM) and Globin-like (a.1, PDB:3ZHW, Lig:HEM). Here similarity is not detectable both at the sequence and the structure, highlighting the use of the method to find commonalities in the site of these proteins. In both the cases, SiteMotif reported the highest number of aligned residues, which was followed by G-LoSA and then by Pocketalign and APoc. (TIF) [file pcbi.1009901.s004.tif]

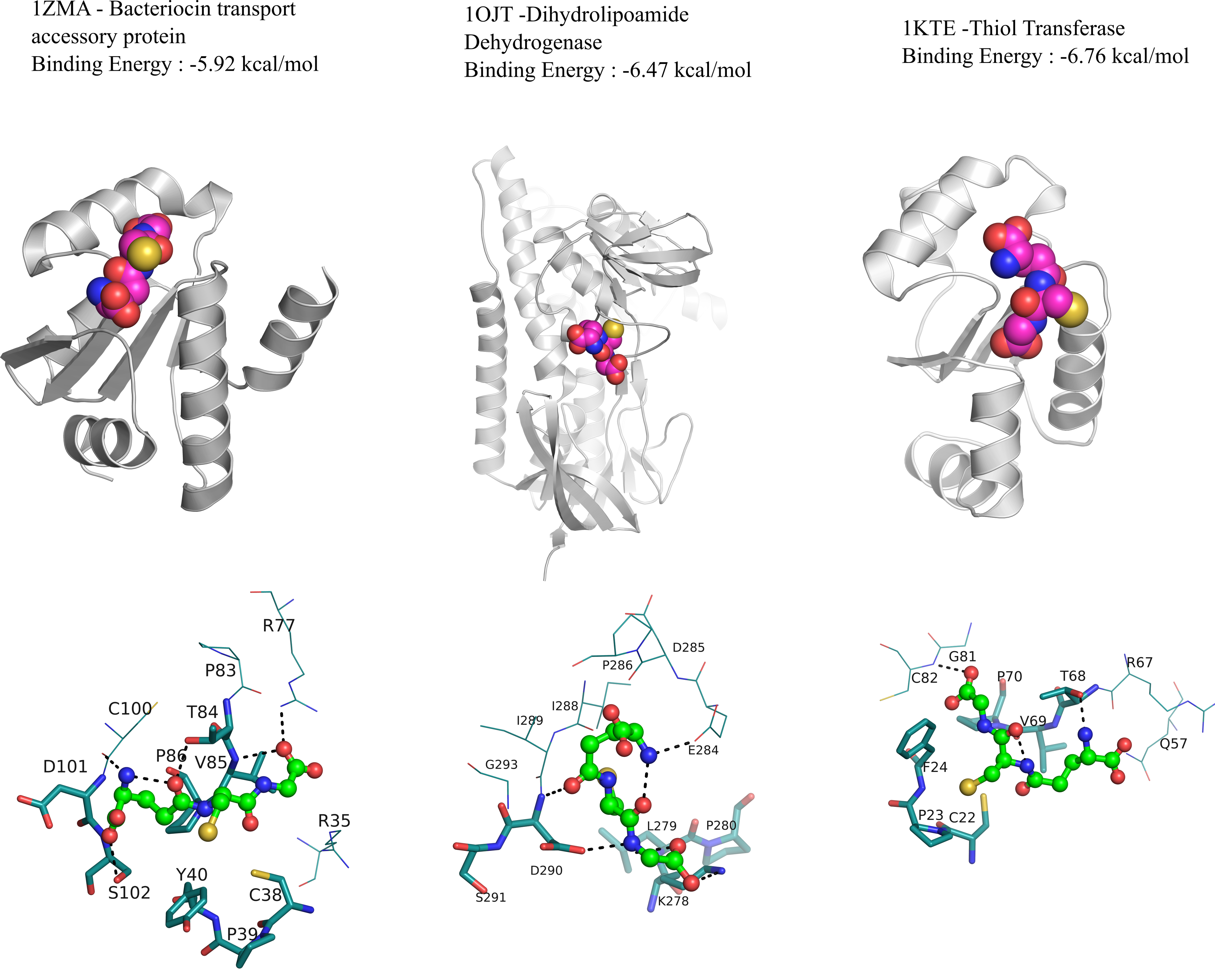

Supplement: S5 Fig — From PocketDB, ligand binding pockets of all proteins are taken and compared with the derived motif for glutathione. Residues present in the binding site in each case are also shown. Glutathione motif residues are shown as thicker lines, while glutathione is in ball-and-stick representation. The software AutoDock was used to calculate theoretical binding affinity between protein and ligand. (TIF) [file pcbi.1009901.s005.tif]

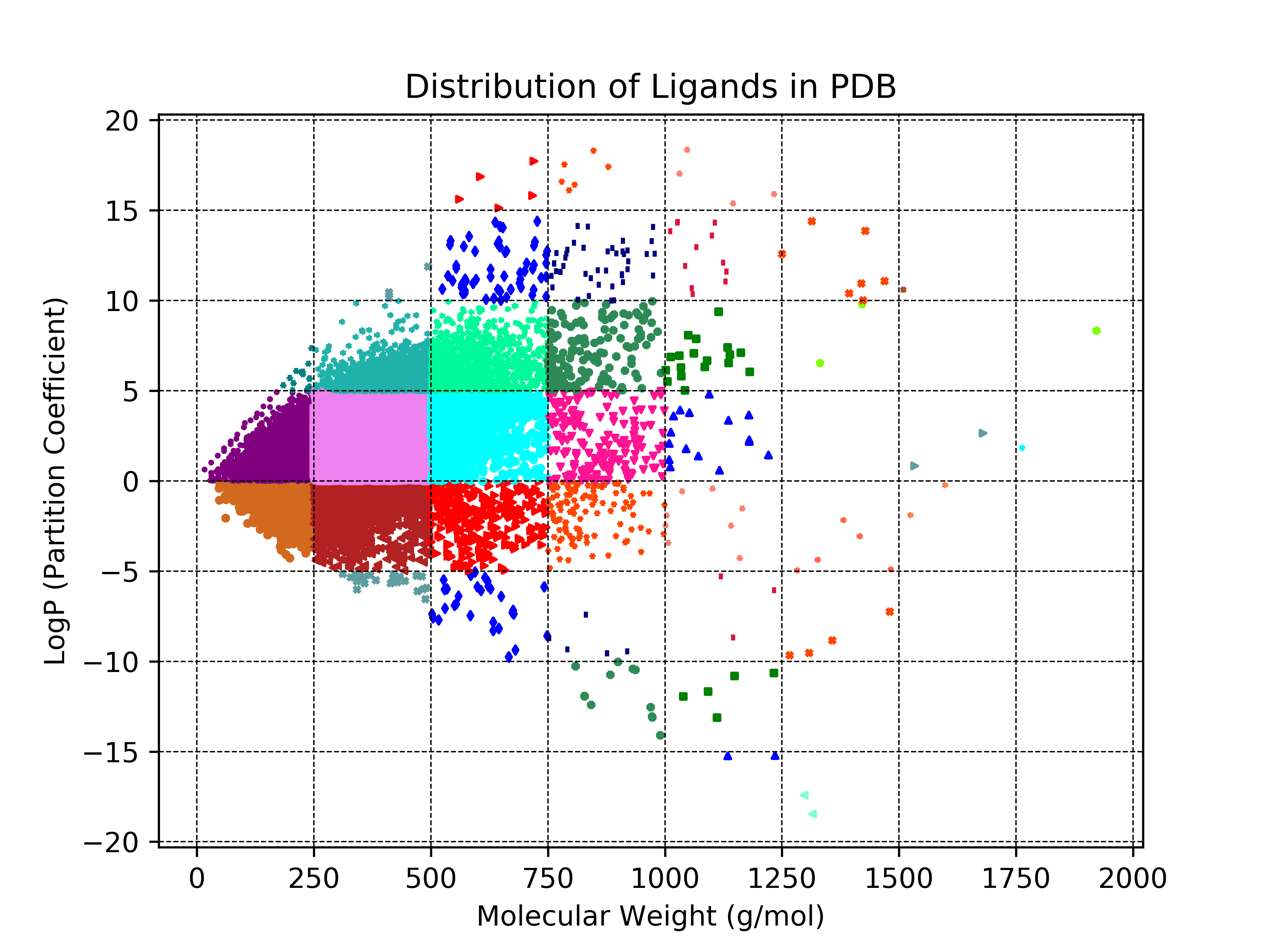

Supplement: S6 Fig — For every ligand, we derived two chemical descriptors: 1) the molecular weight and 2) the partition coefficient. LogP is a direct correlation between ligands and solubility. The higher the LogP the more lipophilic the ligand. From the descriptors, a spaced 2D bin was created, from which 20 distinct ligands, one from each grid, were chosen for sensitivity testing. (TIF) [file pcbi.1009901.s006.tif]
