# Supplementary material for: SiteMotif: A graph-based algorithm for deriving structural motifs in Protein Ligand binding sites
Source: PLoS Comput Biol. 2022 Feb 24;18(2):e1009901. doi: 10.1371/journal.pcbi.1009901 (PMC8903255; doi:10.1371/journal.pcbi.1009901)
Supplement: S1 Table — Superfamily c.37.1 constitutes a total of 15,129 pairs of sites binding to adenosine diphosphate. Similarly, proteins belonging to c.23.5 (FAD binding proteins) possess 3136 pairs of sites, and a.1.1 (Heme binding proteins) comprises 345 site pairs. The binding sites of each of three SCOP superfamilies were taken to test the sensitivity of SiteMotif scores. MPI version of SiteMotif was used to compare 65,789 site pairs which completed the job in 3 hours on an 8 core Linux architecture. For each entry, the conservation index for each residue was computed as the fraction of the number of residues aligned by the total number of residues present. The final column represents the output of site alignment obtained using SiteMotif along with the sequence logo generated using WebLogo. (DOCX) [file pcbi.1009901.s007.docx]

| S. No. | SCOP Family (Ligand Id, Representative PDB, No. of sites, No. of site pairs) | Top Conserved Residues with its conservation index | WebLogo + Site Superposition |
| --- | --- | --- | --- |
| 1 | **c.37.1** (ADP, 1IY1, n=123, N=15,129) | K456(1.0), G455(1.0), S457(0.98), S454(0.98), S458(0.93), S452(0.93), G453(0.89), L451(0.83) | 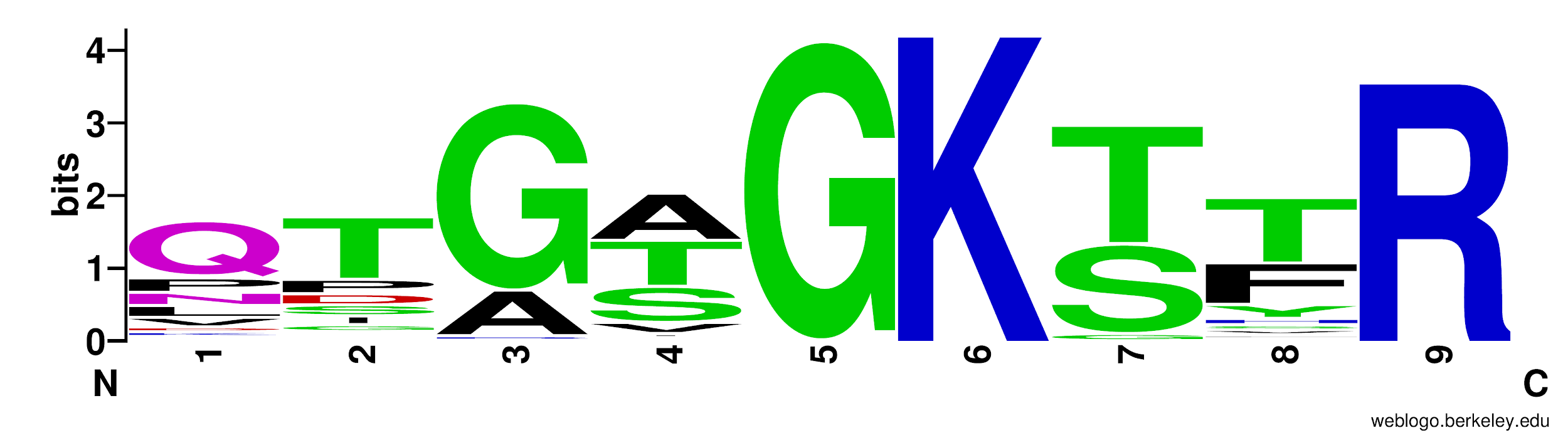  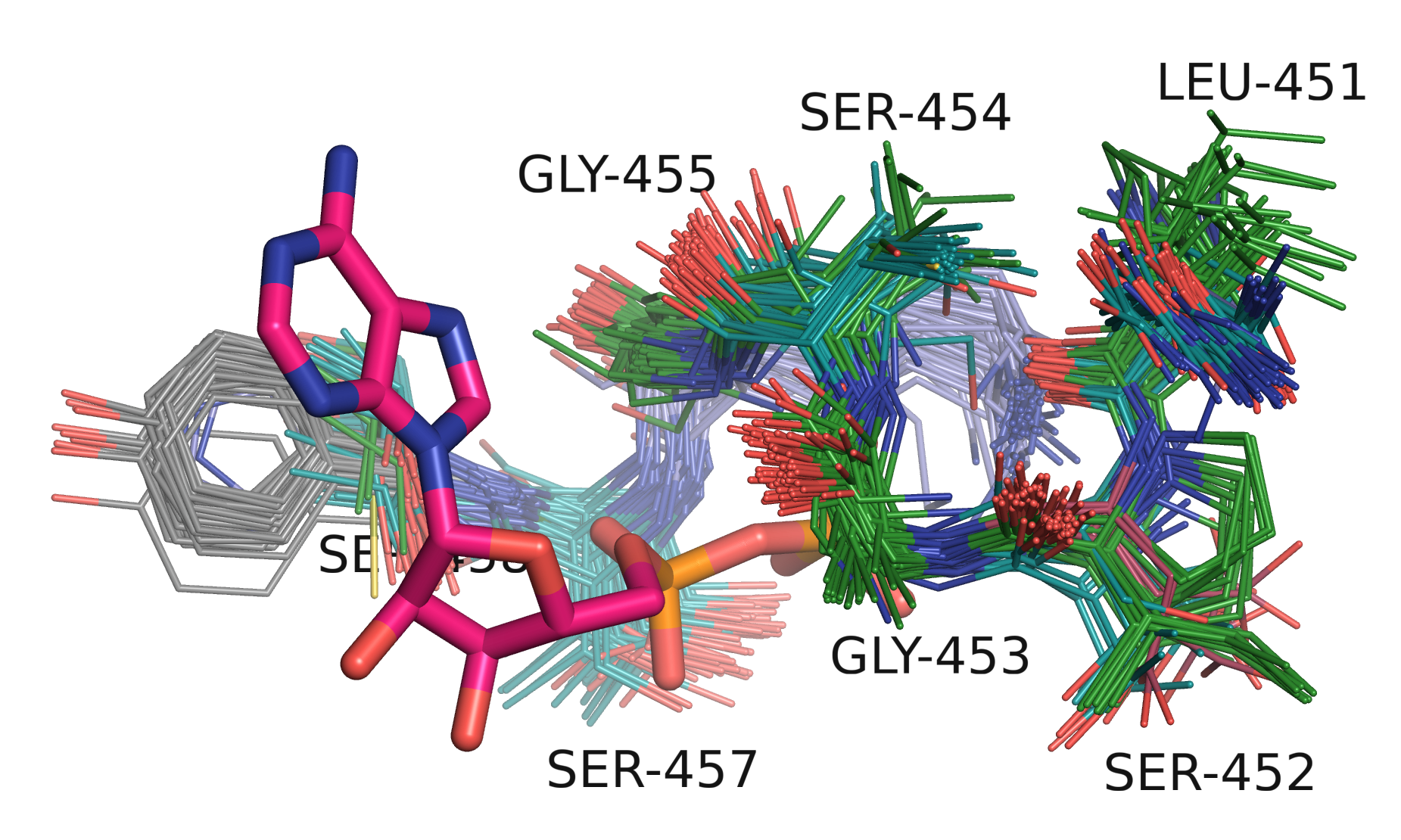 |
| 2 | **c.23.5** (FAD, 2QWX, n=56, N=3,136 ) | L103(1.0), G149(1.0), F106(1.0), N18(1.0), T148(1.0), T147(1.0), A20(1.0), Q104(1.0), R200(1.0), P102(1.0), S16(1.0), W105(1.0), I192(1.0), F17(1.0), H11(1.0), G150(1.0), Y155(0.96), L204(0.8), T15(0.54) | 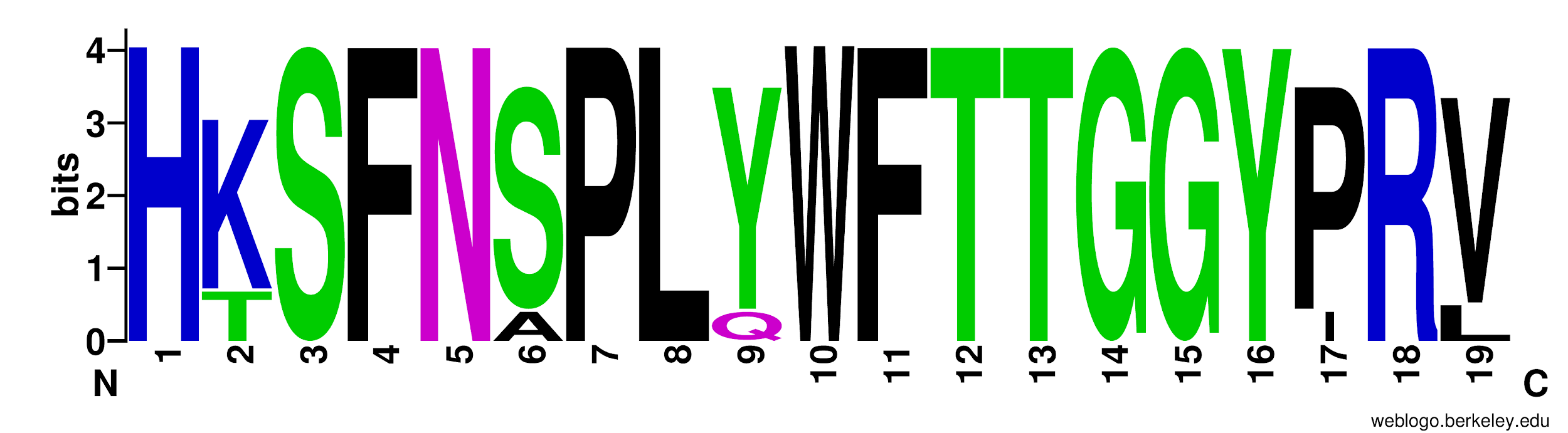  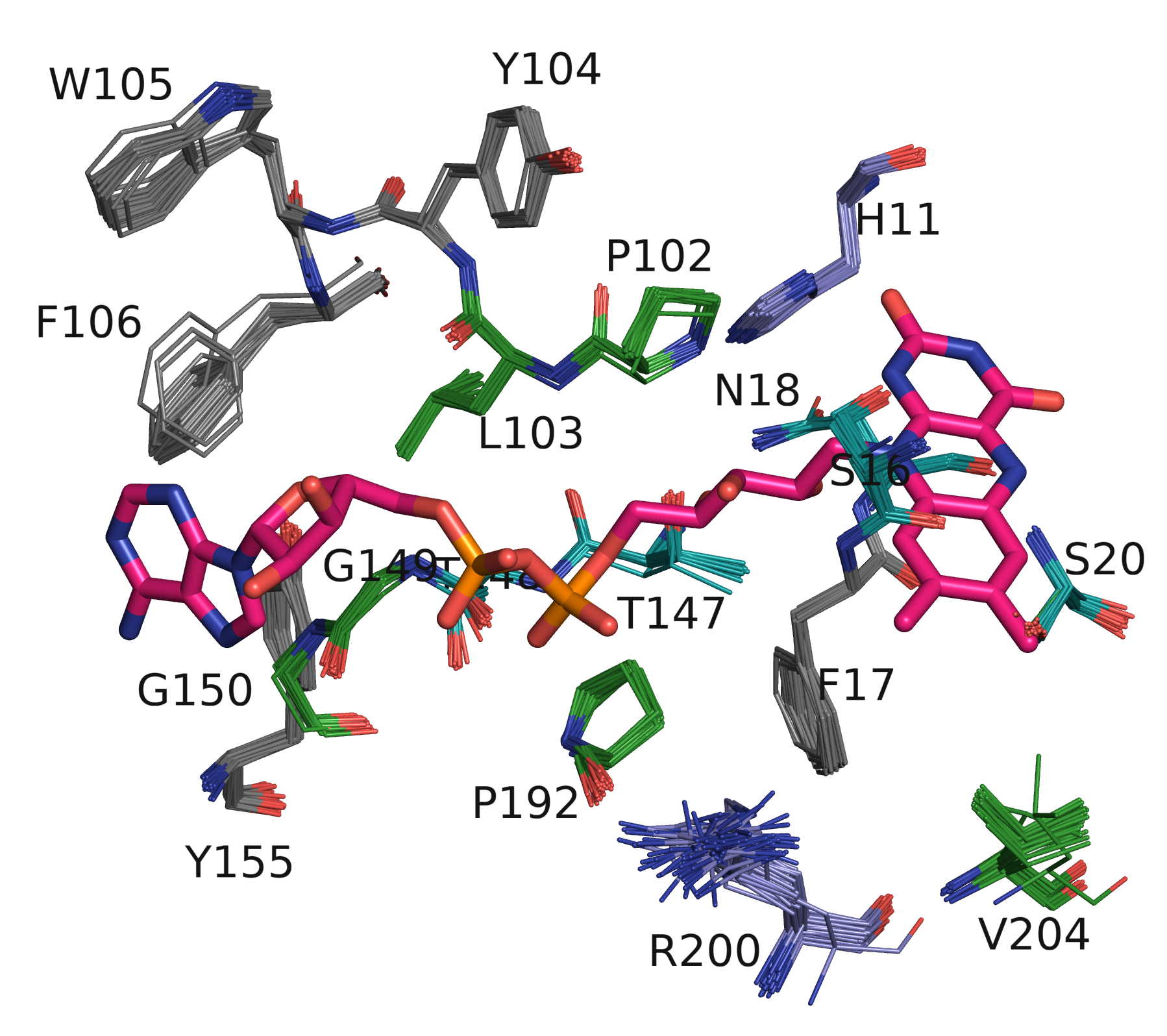 |
| 3 | **a.1.1** (HEM, 1DUO, n=218, N=47,524 ) | K42(0.93), F43(0.91), T39(0.9), I107(0.89), L104(0.88), V68(0.85), Y103(0.84), T67(0.84), I99(0.79), H64(0.76), A71(0.71), L89(0.66), S92(0.65), H97(0.6), L72(0.55) | 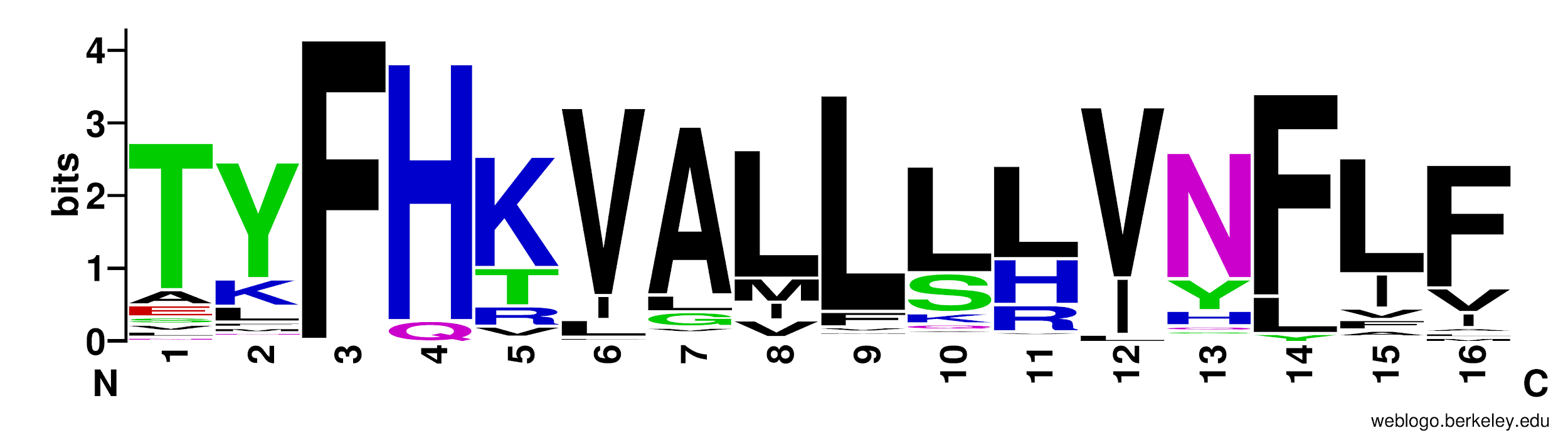  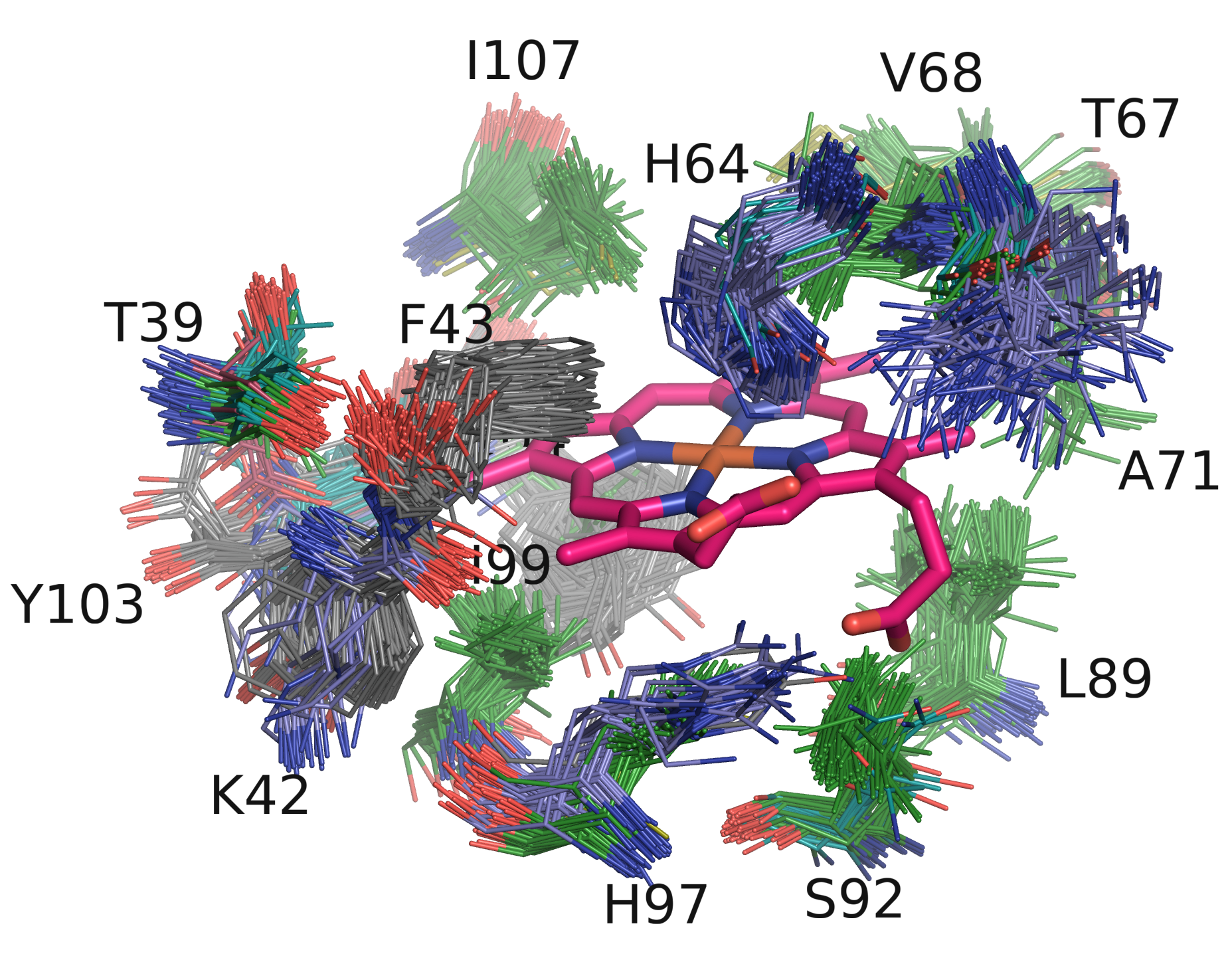 |

Table S1: Assessing the threshold cutoff of M-dist score and the quality of final site alignment associated with three different SCOP superfamilies. Superfamily c.37.1 constitutes a total of 15,129 pairs of sites binding to adenosine diphosphate. Similarly, proteins belonging to c.23.5 (FAD binding proteins) possess 3136 pairs of sites, and a.1.1 (Heme binding proteins) comprises 345 site pairs. The binding sites of each of three SCOP superfamilies were taken to test the sensitivity of SiteMotif scores. MPI version of SiteMotif was used to compare 65,789 site pairs which completed the job in 3 hours on an 8 core Linux architecture. For each entry, the conservation index for each residue was computed as the fraction of the number of residues aligned by the total number of residues present. The final column represents the output of site alignment obtained using SiteMotif along with the sequence logo generated using WebLogo.
