# Supplementary material for: SiteMotif: A graph-based algorithm for deriving structural motifs in Protein Ligand binding sites
Source: PLoS Comput Biol. 2022 Feb 24;18(2):e1009901. doi: 10.1371/journal.pcbi.1009901 (PMC8903255; doi:10.1371/journal.pcbi.1009901)
Supplement: S2 Table — Two predominant SCOP superfamily well reported to recognise cognate ligands was used here. Superfamily S-Adenosyl-L-Methionine-dependent methyltransferases (c.66.1) majorly binds with s-adenosyl methionine (SAM) ligand while P-loop containing nucleoside triphosphate hydrolase (c.37.1) binds to adenosine diphosphate (ADP) molecule. Both SAM binding protein and ADP binding protein sites align well in all structures with an average RMSD of 0.53Å and 0.52Å, respectively. (DOCX) [file pcbi.1009901.s008.docx]

| **Description of Ligand name, representative protein and SCOP superfamily** | **PDB Members** | **SCOP families** | **Calculated Sequence Identity with its representative** | **RMSD of aligned residues** |
| --- | --- | --- | --- | --- |
| **S-Adenosylhomocysteine**  **(SAH), Isoaspartate O-Methyltransferase  (1I1N),  S-Adenosyl-L-Methionine-dependent methyltransferases  (c.66.1)** | 10MH | c.66.1.26 | 1.7 | 0.52 |
|  | 1AV6 | c.66.1.25 | 11.5 | 0.53 |
|  | 1BOO | c.66.1.11 | 5.3 | 0.56 |
|  | 1D2H | c.66.1.5 | 7 | 0.51 |
|  | 1F3L | c.66.1.6 | 10.1 | 0.42 |
|  | 1HNN | c.66.1.15 | 14.2 | 0.6 |
|  | 1I1N | c.66.1.7 | 100 | 0 |
|  | 1JQD | c.66.1.19 | 10.5 | 0.54 |
|  | 1KHH | c.66.1.16 | 9.8 | 0.59 |
|  | 1KPG | c.66.1.18 | 15 | 0.58 |
|  | 1L3I | c.66.1.22 | 17.6 | 0.63 |
|  | 1M6E | c.66.1.35 | 5.2 | 0.36 |
|  | 1NV9 | c.66.1.30 | 8.4 | 0.51 |
|  | 1Q0S | c.66.1.28 | 2.2 | 0.4 |
|  | 1QAN | c.66.1.24 | 15 | 0.62 |
|  | 1RI1 | c.66.1.34 | 11.9 | 0.63 |
|  | 1SUI | c.66.1.1 | 17.1 | 0.42 |
|  | 1U2Z | c.66.1.31 | 10.9 | 0.58 |
|  | 1WY7 | c.66.1.32 | 18.1 | 0.59 |
|  | 1WZN | c.66.1.43 | 13.3 | 0.58 |
|  | 2BR5 | c.66.1.50 | 2.3 | 0.35 |
|  | 2EFJ | c.66.1.0 | 3.8 | 0.7 |
|  | 2EJU | c.66.1.58 | 13.2 | 0.69 |
|  | 2GB4 | c.66.1.36 | 17.5 | 0.62 |
|  | 2H00 | c.66.1.54 | 14.3 | 0.57 |
|  | 2OB2 | c.66.1.37 | 15.2 | 0.53 |
|  | 2WK1 | c.66.1.61 | 15.2 | 0.45 |
|  | 2XVM | c.66.1.44 | 15.6 | 0.53 |
|  | 3GU3 | c.66.1.49 | 11.4 | 0.61 |
|  | 3NDJ | c.66.1.60 | 13.5 | 0.67 |
|  | 5EPE | c.66.1.21 | 18.7 | 0.45 |
| **Adenosine diphosphate (ADP), Pantothenate kinase (1SQ5), P-loop containing nucleoside triphosphate hydrolases (c.37.1)** | 1BG2 | c.37.1.9 | 5.2 | 0.54 |
|  | 1BS1 | c.37.1.10 | 8.9 | 0.43 |
|  | 1E2D | c.37.1.1 | 14 | 0.62 |
|  | 1F3O | c.37.1.12 | 8.4 | 0.78 |
|  | 1G41 | c.37.1.20 | 15.4 | 0.67 |
|  | 1G6O | c.37.1.11 | 14.1 | 0.5 |
|  | 1HTW | c.37.1.18 | 9.1 | 0.55 |
|  | 1L4U | c.37.1.2 | 10.4 | 0.62 |
|  | 1M7G | c.37.1.4 | 17.2 | 0.68 |
|  | 1SQ5 | c.37.1.6 | 100 | 0 |
|  | 1T5C | c.37.1.0 | 15.2 | 0.53 |
|  | 1BG2 | c.37.1.9 | 5.2 | 0.54 |

Table S2: Testing the performance of SiteMotif on protein binding to the same ligand and adopts the same fold but unrelated at the sequence (level-3 dataset). Two predominant SCOP superfamily well reported to recognise cognate ligands was used here. Superfamily S-Adenosyl-L-Methionine-dependent methyltransferases (c.66.1) majorly binds with s-adenosyl methionine (SAM) ligand while P-loop containing nucleoside triphosphate hydrolase (c.37.1) binds to adenosine diphosphate (ADP) molecule. Both SAM binding protein and ADP binding protein sites align well in all structures with an average RMSD of 0.53Å and 0.52Å, respectively.
