# Supplementary material for: SiteMotif: A graph-based algorithm for deriving structural motifs in Protein Ligand binding sites
Source: PLoS Comput Biol. 2022 Feb 24;18(2):e1009901. doi: 10.1371/journal.pcbi.1009901 (PMC8903255; doi:10.1371/journal.pcbi.1009901)
Supplement: S6 File — (PDF) [file pcbi.1009901.s014.pdf]

The following tutorial describes each step necessary to execute the program SiteMotif properly.

For this tutorial, we are going to use the datasets that are already available in our GitHub repository (https://github.com/santhoshgits/MAPP-3D).

SiteMotif is programmed to work with both pair-wise alignment and multiple alignments when given many binding sites. Here we are going to explain both the cases.

Case A) Pair Wise Alignment

Let us consider the binding site of two proteins, 1L2T and 1TF7, for which we need to find if any residues lining their binding sites are similar. 1L2T is Bacterial ABC transporter cassette and 1TF7 is a Circadian clock protein KaiC. Since these two proteins are complexed with same ATP ligand, we want to check if their binding sites are similar.

1L2T

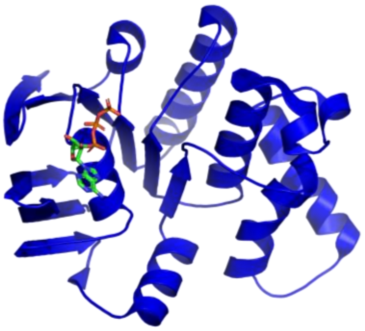

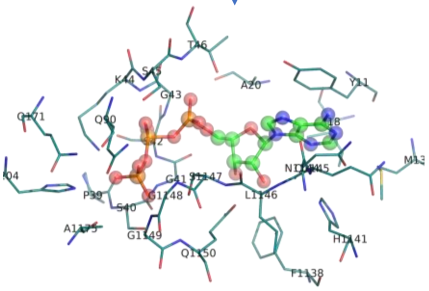

1TF7

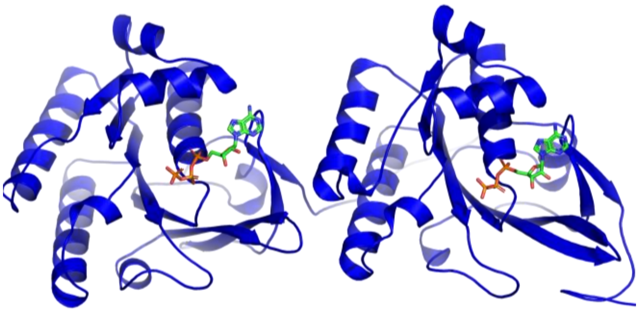

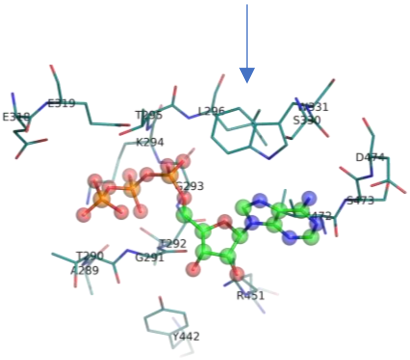

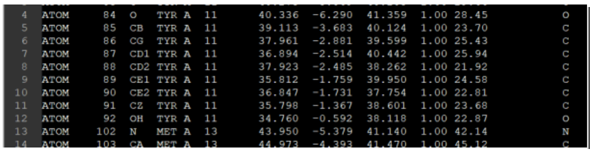

```
ATOM     84  O   TYR A  11      40.336  -6.290  41.359  1.00 28.45           O
ATOM     85  CB  TYR A  11      39.113  -3.683  40.124  1.00 23.70           C
ATOM     86  CG  TYR A  11      37.961  -2.881  39.599  1.00 25.43           C
ATOM     87  CD1 TYR A  11      36.894  -2.514  40.442  1.00 25.94           C
ATOM     88  CD2 TYR A  11      37.923  -2.485  38.262  1.00 21.92           C
ATOM     89  CE1 TYR A  11      35.812  -1.759  39.950  1.00 24.58           C
ATOM     90  CE2 TYR A  11      36.847  -1.731  37.754  1.00 22.81           C
ATOM     91  CZ  TYR A  11      35.798  -1.367  38.601  1.00 23.68           C
ATOM     92  OH  TYR A  11      34.760  -0.592  38.118  1.00 22.87           O
ATOM    102  N   MET A  13      43.950  -5.379  41.140  1.00 42.14           N
ATOM    103  CA  MET A  13      44.973  -4.393  41.470  1.00 45.12           C
```

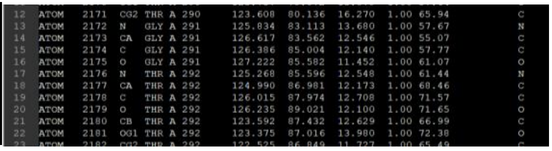

```
ATOM   2171  CG2 THR A 290     123.608  80.136  16.270  1.00 65.94           C
ATOM   2172  N   GLY A 291     125.834  83.113  13.680  1.00 57.67           N
ATOM   2173  CA  GLY A 291     126.617  83.562  12.546  1.00 55.07           C
ATOM   2174  C   GLY A 291     126.386  85.004  12.140  1.00 57.77           C
ATOM   2175  O   GLY A 291     127.222  85.582  11.452  1.00 61.07           O
ATOM   2176  N   THR A 292     125.268  85.596  12.548  1.00 61.44           N
ATOM   2177  CA  THR A 292     124.990  86.981  12.173  1.00 68.46           C
ATOM   2178  C   THR A 292     126.015  87.974  12.708  1.00 71.57           C
ATOM   2179  O   THR A 292     126.235  89.021  12.100  1.00 71.65           O
ATOM   2180  CB  THR A 292     123.592  87.432  12.629  1.00 66.99           C
ATOM   2181  OG1 THR A 292     123.375  87.016  13.980  1.00 72.38           O
ATOM   2182  CG2 THR A 292     122.525  86.849  11.727  1.00 65.49           C
```

NOTE: SiteMotif only require binding site coordinates as the input file. If a user has a coordinate of entire protein, then he/she is first required to find binding site residues using some pocket prediction algorithm or from a bound ligand pick all residues whose atoms are present with 4.5A of ligand atom. After that, The ATOM coordinates of predicted binding site residues can be saved into a new file ‘site.pdb’.

To run a pair-wise comparison, we only need one python file ‘pocket_matrix7.py’. What this file does?

i) It constructs a distance matrix using the all-pair distance difference computed using the Ca, Cb and Centroid of each amino acids
ii) Enables a Graph traversal search and stores all paths of length greater than 4.
iii) For each identified paths, a least square alignment based on Kabsch algorithm is carried out to find the most optimal alignment.

Input: pocket_matrix7.py requires two inputs (site1.pdb and site2.pdb).

For the example inputs, it would be ‘python pocket_matrix7.py 1L2T_site.pdb 1TF7_site.pdb’. By default, second site is considered as fixed protein, first site (reference site) will be super imposed onto the second one. For this example, it is 1L2T that is aligned onto 1TF7.

Output: Pair-wise execution generates five file in addition to a terminal output.

1. align.txt - contains list of residues matched between sites
2. fixed.pdb - translated coordinate of the fixed binding site
3. frag.pdb - translated coordinate of the reference site
4. site1.pdb - same as fixed.pdb but contains only the coordinates of matched residues
5. site2.pdb - same as frag.pdb but contains only the coordinates of matched residues

Scores = 10/26, 10/25, 0.4, 0.39, 0.65 (No.of.ResidueAligned/length of site1, No.of.ResidueAligned/length of site2, $Mdist_{min}$, $Mdist_{max}$, Mseq).

Interpretation: The above pairs have an $Mdist_{max}$ of 0.39 and $Mdist_{min}$ of 0.4. This is expected as the number of residues is 25 and 26. However the number of residues that is aligned is 10. Hence pay attention to length of alignment as well, as SiteMotif is capable even to capture the smallest alignment that exist between two binding sites.

Visualising the alignment of site1.pdb and site2.pdb in pymol.

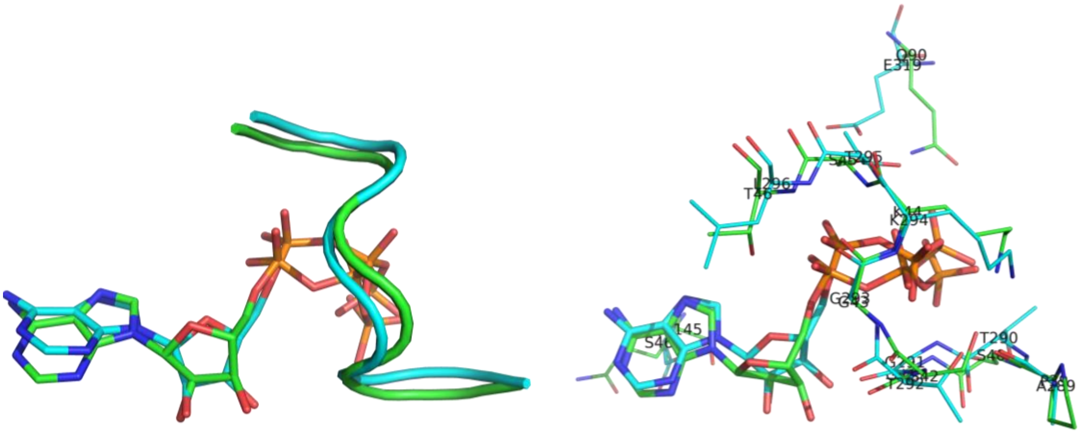

Case B) Multiple Alignments

Multiple alignments are especially useful when we want to carry out alignment for large number of pairs (typically in 1000's or million pairs). As the number of pairs are going to be large, we have created an MPI version of SiteMotif that is suitable to handle exhaustive comparisons.

Please refer to README.md from https://github.com/santhoshgits/MAPP-3D to setup MPI library for your machine.

For the purpose of this exercise, we are going to take 100 ATP binding site and check if we could identify any motif. This main script that is going to execute parallel version of SiteMotif is 'pocket_matrix_mpi7.py'.

Steps:

1) Create a folder 'ATP_site' and copy all your input binding sites to the folder.
2) Run the script Pairs.py. This will generate a tab separated pairs. For 100 sites, we will get 10,000 Pairs.
3) Run the script PDBSize.py. This will get the residue number of all PDB structures.

Inputs: mpirun -n 4 python pocket_matrix_7.py <Argument1> < Argument2> < Argument3>

Argument1 - ATP site folder
Argument2 - output of Pairs.py
Argument3 - output of PDBSize.py

Output: align_output.txt

To find representative for the ATP binding site, any clustering algorithm can be used. Here we pick one representative based on number connections with the other ATP sites after imposing a cut-off of M-$dist_{max}$ > 0.4.

If the extent of similarity is very minimum, it is completely good to cluster sites based on the number of residue match.

We have provided two additional scripts i) Analyse.py (To find the representative sites) and ii) Motif.py (To generate Motif). This is not a part of SiteMotif program and hence users are advised to analyse the result file (align_output.txt) according to their needs.

Command to run Analyse.py:

Python Analyse.py align_output.txt .4 4

Output: 1VCI. This protein shares maximum contact with other ATP protein for the specified threshold.

Command to run Motif.py:

Python Motif.py align_output.txt 1VCI_ATP_A_374.pdb 4

Output: [FLWK]-x-x-[HFR]-x-S-x(17,18)-P-[TS]-G-S-G-K-[TS]
